# Supplementary material for: Phosphine Oxide-Promoted Rh(I)-Catalyzed C–H Cyclization of Benzimidazoles with Alkenes
Source: Molecules. 2023 Jan 11;28(2):736. doi: 10.3390/molecules28020736 (PMC9864171; doi:10.3390/molecules28020736)
Supplement: Supplementary file 1 [file molecules-28-00736-s001.zip › molecules-2095821-supplementary.pdf]

Supplementary Information

Phosphine Oxide-Promoted Rh(I)-Catalyzed C–H Cyclization of Benzimidazoles with Alkenes

Mingzhen Lu [1,†] Weiwei Xu [1,†] and Mengchun Ye [1,2]*

Contents

1. General Information 2
2. Typical Procedure for Rh-Catalyzed C–H Cyclization 2
3. Procedure for Enantioselective Rh/Co-Catalyzed C–H Cyclization 5
4. Procedure for Mechanistic Experiments 6
5. Reference 7
6. NMR Spectra of Products 8
7. HPLC Charts of Chiral Products 21

1. General Information

Unless stated otherwise, all reactions were conducted under N_2 atmosphere. All solvents were received from commercial sources without further purification. Commercially available reagents were used as received. Non-commercially available substrates were synthesized following reported protocols. Thin-layer chromatography (TLC) was performed by UV absorbance (254 nm). 200–300 mesh silica gel was used for column chromatography separation. NMR spectra were recorded on Bruker AV 400 spectrometer at 400 MHz (^{1}H NMR), 100 MHz (^{13}C NMR), 376 MHz (^{19}F NMR) and 162 MHz (^{31}P NMR). Proton and carbon chemical shifts are reported relative to the solvent used as an internal reference ($CDCl_3$: δ_H = 7.26 ppm; δ_C = 77.16 ppm). All coupling constants (*J* values) were reported in Hertz (Hz). Multiplicities are reported as follows: singlet (s), doublet (d), doublet of doublets (dd), triplet (t), triplet of doublets (td), quartet (q), and multiplet (m). Chiral high-performance liquid chromatography (HPLC) analysis was performed using an Agilent 1260 with commercial ChiralPak 4.6 × 250 mm columns. Optical rotations were determined by a Rudolph Autopol VI polarimeter. Substrate[1] and ligand[1,2] was prepared according to our previous work.

2. Typical Procedure for Rh-Catalyzed C–H Cyclization

To a 15 mL oven dried tube were added ligand Mes-DAPO (6.8 mg, 10 mol%), $[Rh(cod)_2]BF_4$ (8.1 mg, 10 mol%), benzimidazole derivative (0.2 mmol, 1eq.) and dry degassed toluene (2.0 mL) under N_2 atmosphere. Then $AlMe_2Cl$ (1.0 M/hexane, 40 µL, 20 mol%) was added and the tube was sealed. After heated at 120 °C for 3 h, the mixture was cooled to r.t., quenched with 2 mL of 5% EDTA disodium salt solution, filtered through a short plug of silica gel (EtOAc as the eluent) and concentrated in vacuo to afford a crude product. Further purification by flash column chromatography on silica gel (eluting with EtOAc/hexanes) gave the pure product.

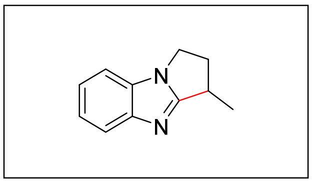

3-Methyl-2,3-dihydro-1*H*-benzo[*d*]pyrrolo[1,2-a]imidazole (2a)
White solid (96% yield). **^{1}H NMR** (400 MHz, $CDCl_3$) δ 7.79 – 7.64 (m, 1H), 7.31 – 7.16 (m, 3H), 4.18 – 4.07 (m, 1H), 4.06 – 3.91 (m, 1H), 3.46 – 3.27 (m, 1H), 2.94 – 2.81 (m, 1H), 2.32 – 2.22 (m, 1H), 1.48 (d, *J* = 7.0 Hz, 3H). **^{13}C NMR** (100 MHz, $CDCl_3$) δ 164.5, 148.7, 132.4, 121.9, 121.7, 119.7, 109.6, 42.0, 35.4, 31.0, 18.1.

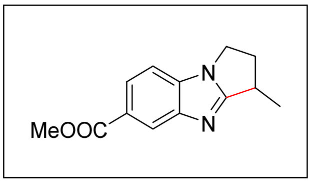

Methyl-3-methyl-2,3-dihydro-1*H*-benzo[d]pyrrolo[1,2-a]imidazole-6-carboxylate (2b)
White solid (94% yield). **^{1}H NMR** (400 MHz, $CDCl_3$) δ 8.39 (s, 1H), 7.93 (d, *J* = 8.4 Hz, 1H), 7.27 (d, *J* = 8.3 Hz, 1H), 4.18 – 4.11 (m, 1H), 4.05 – 3.97 (m, 1H), 3.91 (s, 3H), 3.43 – 3.31 (m, 1H), 2.96

– 2.85 (m, 1H), 2.35 – 2.24 (m, 1H), 1.48 (d, *J* = 7.0 Hz, 3H). **^{13}C NMR** (100 MHz, $CDCl_3$) *δ* 167.9, 166.3, 148.4, 135.7, 123.8, 122.0, 109.2 , 77.5, 52.1, 42.1, 35.4, 31.1, 18.0.

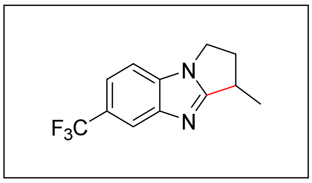

3-Methyl-6-(trifluoromethyl)-2,3-dihydro-1*H*-benzo[*d*]pyrrolo[1,2-a]imidazole (2c)
White solid (97% yield). **^{1}H NMR** (400 MHz, $CDCl_3$) *δ* 7.96 (s, 1H), 7.45 (d, *J* = 8.4 Hz, 1H), 7.35 (d, *J* = 8.4 Hz, 1H), 4.24 – 4.12 (m, 1H), 4.12 – 3.99 (m, 1H), 3.49 – 3.34 (m, 1H), 2.99 – 2.87 (m, 1H), 2.38 – 2.27 (m, 1H), 1.50 (d, *J* = 7.1 Hz, 3H). **^{13}C NMR** (100 MHz, $CDCl_3$) *δ* 166.6, 148.1, 134.4, 126.4, 124.4, 124.1, 123.7, 119.1, 119.0, 117.3, 117. 3, 109.9, 42.2, 35.4, 31.2, 18.0. **^{19}F NMR** (376 MHz, $CDCl_3$) *δ* -60.5.

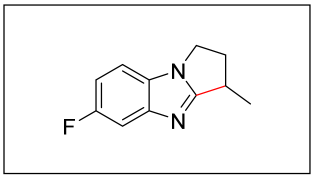

6-Fluoro-3-methyl-2,3-dihydro-1*H*-benzo[*d*]pyrrolo[1,2-a]imidazole (2d)
White solid (96% yield). **^{1}H NMR** (400 MHz, $CDCl_3$) *δ* 7.36 (d, *J* = 9.7 Hz, 1H), 7.16 (dd, *J* = 8.7, 4.6 Hz, 1H), 6.99 – 6.85 (m, 1H), 4.15 – 4.05 (m, 1H), 4.02 – 3.92 (m, 1H), 3.41 – 3.25 (m, 1H), 2.95 – 2.80 (m, 1H), 2.32 – 2.21 (m, 1H), 1.46 (d, *J* = 7.0 Hz, 3H). **^{13}C NMR** (100 MHz, $CDCl_3$) *δ* 166.0, 160.3, 158.0, 158.0, 149.1, 149.0, 129.0, 110.1, 109.8, 109.7, 109.6, 105.8, 105.5, 42.2, 35.4, 31.3, 18.0. **^{19}F NMR** (376 MHz, $CDCl_3$) *δ* -121.6.

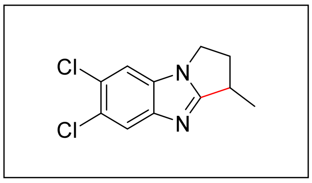

6,7-Dichloro-3-methyl-2,3-dihydro-1*H*-benzo[*d*]pyrrolo[1,2-a]imidazole (2e)
White solid (97% yield). **^{1}H NMR** (400 MHz, $CDCl_3$) *δ* 7.72 (s, 1H), 7.32 (s, 1H), 4.12 – 4.01 (m, 1H), 4.00 – 3.88 (m, 1H), 3.40 – 3.26 (m, 1H), 2.95 – 2.82 (m, 1H), 2.34 – 2.22 (m, 1H), 1.45 (d, *J* = 7.2 Hz, 3H). **^{13}C NMR** (100 MHz, $CDCl_3$) *δ* 166.7, 148.0, 131.6, 125.7, 125.6, 120.7, 110.9, 42.2, 35.3, 31.1, 17.9.

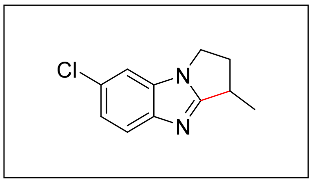

7-Chloro-3-methyl-2,3-dihydro-1*H*-benzo[*d*]pyrrolo[1,2-a]imidazole (2f)
White solid (96% yield). **^{1}H NMR** (400 MHz, $CDCl_3$) *δ* 7.58 (d, *J* = 8.3 Hz, 1H), 7.27 (s, 1H), 7.15 (d, *J* = 8.1 Hz, 1H), 4.28 – 4.07 (m, 1H), 4.07 – 3.90 (m, 1H), 3.58 – 3.24 (m, 1H), 3.09 – 2.78 (m, 1H), 2.44 – 2.19 (m, 1H), 1.47 (d, *J* = 6.1 Hz, 3H). **^{13}C NMR** (100 MHz, $CDCl_3$) *δ* 165.4, 147.2, 132.9, 127.6, 122.3, 120.4, 109.8, 42.1, 35.3, 31.0, 18.0.

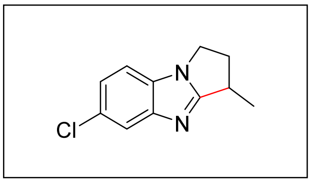

6-Chloro-3-methyl-2,3-dihydro-1*H*-benzo[*d*]pyrrolo[1,2-a]imidazole (2g)
White solid (93% yield). **^{1}H NMR** (400 MHz, $CDCl_3$) δ 7.76 (s, 1H), 7.35 – 7.22 (m, 2H), 4.26 – 4.17 (m, 1H), 4.14 – 4.02 (m, 1H), 3.56 – 3.36 (m, 1H), 3.04 – 2.94 (m, 1H), 2.47 – 2.34 (m, 1H), 1.57 (d, J = 7.0 Hz, 3H). **^{13}C NMR** (100 MHz, $CDCl_3$) δ 165.9, 149.3, 131.0, 127.4, 122.4, 119.5, 110.3, 42.2, 35.3, 31.2, 18.0.

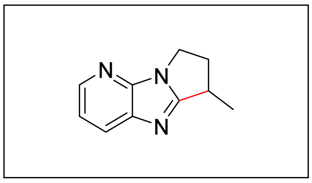

6-Methyl-7,8-dihydro-6*H*-pyrrolo[2',1':2,3]imidazo[4,5-*b*]pyridine (2h)
White solid (95% yield). **^{1}H NMR** (400 MHz, $CDCl_3$) δ 8.25 (d, J = 4.8 Hz, 1H), 7.93 (d, J = 8.0 Hz, 1H), 7.15 (dd, J = 8.0, 4.9 Hz, 1H), 4.34 – 4.24 (m, 1H), 4.16 – 4.06 (m, 1H), 3.50 – 3.28 (m, 1H), 2.99 – 2.81 (m, 1H), 2.35 – 2.25 (m, 1H), 1.49 (d, J = 7.0 Hz, 3H). **^{13}C NMR** (100 MHz, $CDCl_3$) δ 165.8, 146.1, 143.0, 140.8, 127.1, 117.9, 41.5, 35.3, 31.6, 17.8.

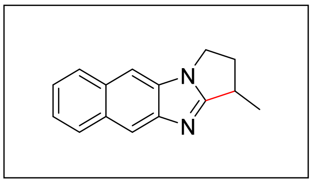

3-Methyl-2,3-dihydro-1*H*-naphtho[2,3-*d*]pyrrolo[1,2-a]imidazole (2i)
White solid (96% yield). **^{1}H NMR** (400 MHz, $CDCl_3$) δ 8.15 (s, 1H), 7.96 (s, 1H), 7.88 (s, 1H), 7.62 (s, 1H), 7.43 – 7.31 (m, 2H), 4.18 – 4.06 (m, 1H), 4.05 – 3.93 (m, 1H), 3.46 – 3.30 (m, 1H), 2.94 – 2.81 (m, 1H), 2.36 – 2.23 (m, 1H), 1.56 – 1.44 (m, 3H). **^{13}C NMR** (100 MHz, $CDCl_3$) δ 168.8, 149.0, 133.3, 130.2, 128.6, 127.4, 124.2, 123.4, 116.3, 105.2, 42.0, 35.3, 31.3, 17.9.

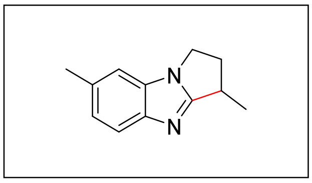

3,7-Dimethyl-2,3-dihydro-1*H*-benzo[*d*]pyrrolo[1,2-a]imidazole (2j)
White solid (61% yield). **^{1}H NMR** (400 MHz, $CDCl_3$) δ 7.57 (d, J = 8.2 Hz, 1H), 7.08 (s, 1H), 7.02 (d, J = 8.3 Hz, 1H), 4.14 – 4.02 (m, 1H), 4.01 – 3.88 (m, 1H), 3.43 – 3.24 (m, 1H), 2.91 – 2.81 (m, 1H), 2.46 (s, 3H), 2.31 – 2.19 (m, 1H), 1.46 (d, J = 7.0 Hz, 3H). **^{13}C NMR** (100 MHz, $CDCl_3$) δ 164.0, 146.5, 132.5, 131.8, 123.2, 119.1, 109.6, 41.8, 35.4, 31.0, 21.8, 18.1.

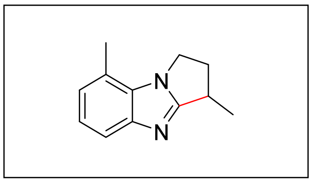

3,8-Dimethyl-2,3-dihydro-1*H*-benzo[*d*]pyrrolo[1,2-a]imidazole (2k)
White solid (52% yield). **^{1}H NMR** (400 MHz, $CDCl_3$) *δ* 7.52 (d, *J* = 8.0 Hz, 1H), 7.07 (t, *J* = 7.6 Hz, 1H), 6.92 (d, *J* = 7.1 Hz, 1H), 4.47 – 4.29 (m, 1H), 4.27 – 4.10 (m, 1H), 3.39 – 3.18 (m, 1H), 2.91 – 2.80 (m, 1H), 2.57 (s, 3H), 2.33 – 2.16 (m, 1H), 1.46 (d, *J* = 7.0 Hz, 3H). **^{13}C NMR** (100 MHz, $CDCl_3$) *δ* 164.3, 148.3, 131.8, 123.2, 121.9, 120.7, 117.3, 44.2, 35.5, 30.6, 18.1, 17.1.

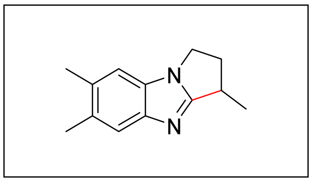

3,6,7-Trimethyl-2,3-dihydro-1*H*-benzo[*d*]pyrrolo[1,2-a]imidazole (2m)
White solid (32% yield). **^{1}H NMR** (400 MHz, $CDCl_3$) *δ* 7.44 (s, 1H), 7.02 (s, 1H), 4.07 – 3.98 (m, 1H), 3.93 – 3.84 (m, 1H), 3.34 – 3.22 (m, 1H), 2.87 – 2.76 (m, 1H), 2.33 (s, 6H), 2.27 – 2.15 (m, 1H), 1.43 (d, *J* = 6.9 Hz, 3H). **^{13}C NMR** (100 MHz, $CDCl_3$) *δ* 163.6, 147.0, 130.8, 130.7, 130.3, 119.7, 109.9, 109.9, 41.8, 35.4, 30.9, 20.4, 20.3, 18.1.

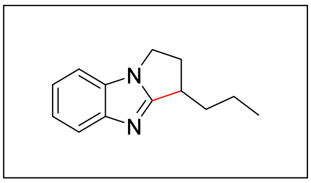

3-Propyl-2,3-dihydro-1*H*-benzo[*d*]pyrrolo[1,2-a]imidazole (2n)
White solid (53% yield). **^{1}H NMR** (400 MHz, $CDCl_3$) *δ* 7.80 – 7.66 (m, 1H), 7.32 – 7.27 (m, 1H), 7.24 – 7.16 (m, 2H), 4.17 – 4.08 (m, 1H), 4.06 – 3.93 (m, 1H), 3.39 – 3.17 (m, 1H), 2.91 – 2.76 (m, 1H), 2.38 – 2.29 (m, 1H), 2.05 – 1.95 (m, 1H), 1.66 – 1.49 (m, 3H), 0.99 (t, J = 7.0 Hz, 3H). **^{13}C NMR** (100 MHz, $CDCl_3$) *δ* 164.0, 148.7, 132.3, 121.9, 121.7, 119.8, 109.6, 42.1, 36.1, 35.5, 33.3, 20.7, 14.3.

3. Procedure for Enantioselective Rh/Co-Catalyzed C–H Cyclization

To a 15 mL oven dried tube were added ligand **L1** (11.1 mg, 10 mol%), $[Rh(cod)_2]BF_4$ (8.1 mg, 10 mol%), benzimidazole derivative (0.2 mmol, 1eq.) and dry degassed toluene (2.0 mL) under N_2 atmosphere. Then $AlMe_2Cl$ (1.0 M/hexane, 40 μL, 20 mol%) was added and the tube was sealed. After heated at 120 °C for 3 h, the mixture was cooled to r.t., quenched with 2 mL of 5% EDTA disodium salt solution, filtered through a short plug of silica gel (EtOAc as the eluent) and concentrated in vacuo to afford a crude product. Further purification by flash column chromatography on silica gel (eluting with EtOAc/hexanes) gave the pure product.

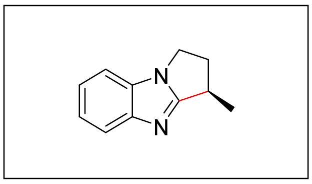

(*R*)-3-Methyl-2,3-dihydro-1*H*-benzo[*d*]pyrrolo[1,2-a]imidazole
White solid (38% yield). **HPLC condition**: Chiralpak IC column, *n*-hexane/*i*-PrOH = 85:15, 1.0 mL/min, 254 nm, $t_{r\text{-major}}$ = 15.6 min, 27% ee. $[\alpha]_D^{28}$ = +2.64 (c 0.5, $CHCl_3$).

To a 15 mL oven dried tube were added ligand **L2** (10.8 mg, 10 mol%), PCy_3 (5.6 mg, 10 mol%), $CoCl_2$ (2.6 mg, 10 mol%), Zn (6.5mg, 50 mol%), benzimidazole derivative (0.2 mmol, 1eq.) and dry degassed toluene (2.0 mL) under N_2 atmosphere. Then $AlMe_3$ (1.0 M/hexane, 80 µL, 40 mol%) was added and the tube was sealed. After heated at 120 °C for 12 h, the mixture was cooled to r.t., quenched with 2 mL of 5% EDTA disodium salt solution, filtered through a short plug of silica gel (EtOAc as the eluent) and concentrated in vacuo to afford a crude product. Further purification by flash column chromatography on silica gel (eluting with EtOAc/hexanes) gave the pure product.

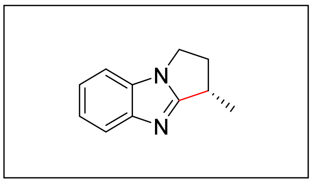

(S)-3-Methyl-2,3-dihydro-1*H*-benzo[*d*]pyrrolo[1,2-a]imidazole
White solid (65% yield). **HPLC condition**: Chiralpak IC column, *n*-hexane/*i*-PrOH = 85:15, 1.0 mL/min, 254 nm, $t_{r\text{-major}}$ = 22.1 min, 45% ee. $[\alpha]_D^{28}$ = -6.80 (c 0.5, $CHCl_3$).

4. Procedure for Mechanistic Experiments

Parallel reactions were set up following the general procedure by using 1a and 1a-d as substrate respectively. Aliquots were taken at intervals for the first 15 minutes. Product yield was determined by ^{1}H NMR using CH_2Br_2 as an internal standard. Data points represent the average of two runs.

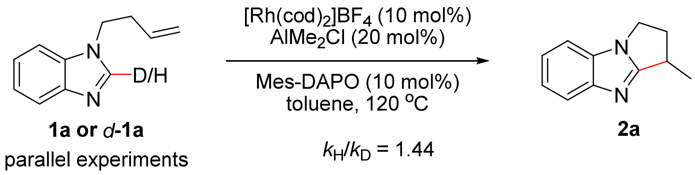

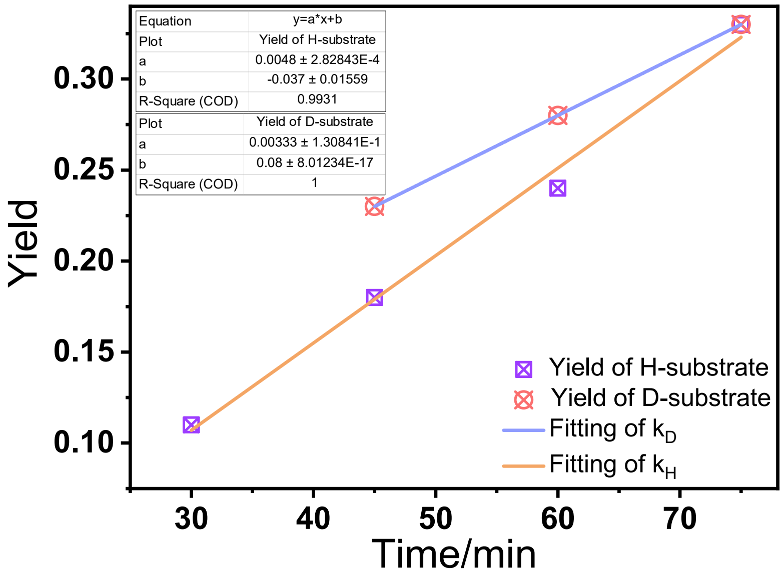

Deuerium-labeling reaction was set up following the general procedure by using *d*-**1a** as the substrate.

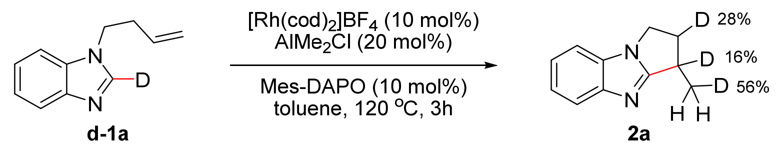

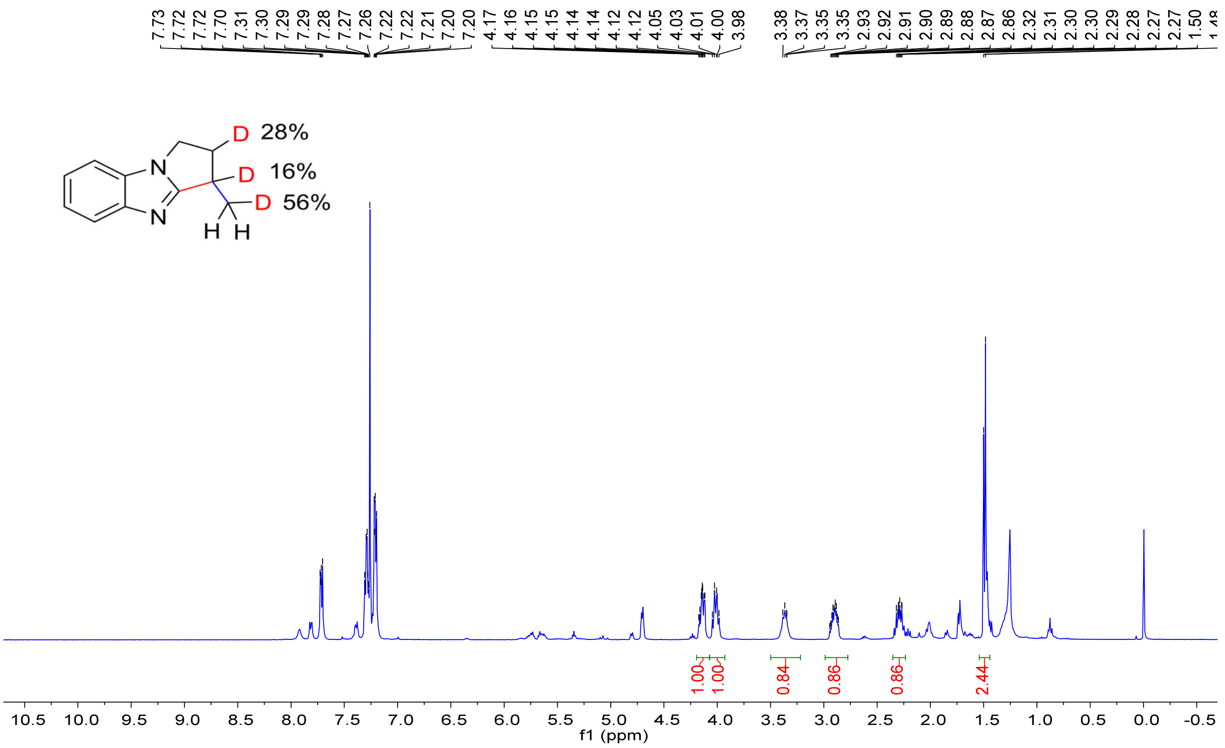

5. Reference

1. Wang, Y.-X.; Qi, S.-L.; Luan, X.-Y.; Han, X.-W.; Wang, S.; Chen, H.; Ye, M. *J. Am. Chem. Soc.* 2018, **140**, *16*, 5360–5364.

2. Chen, H.; Wang, Y.-X.; Luan, Y.-X.; Ye, M. Enantioselective twofold C−H annulation of formamides and alkynes without built-in chelating groups. *Angew. Chem., Int. Ed.* **2020**, *59*, 9428−9432.

6. NMR Spectra of Products

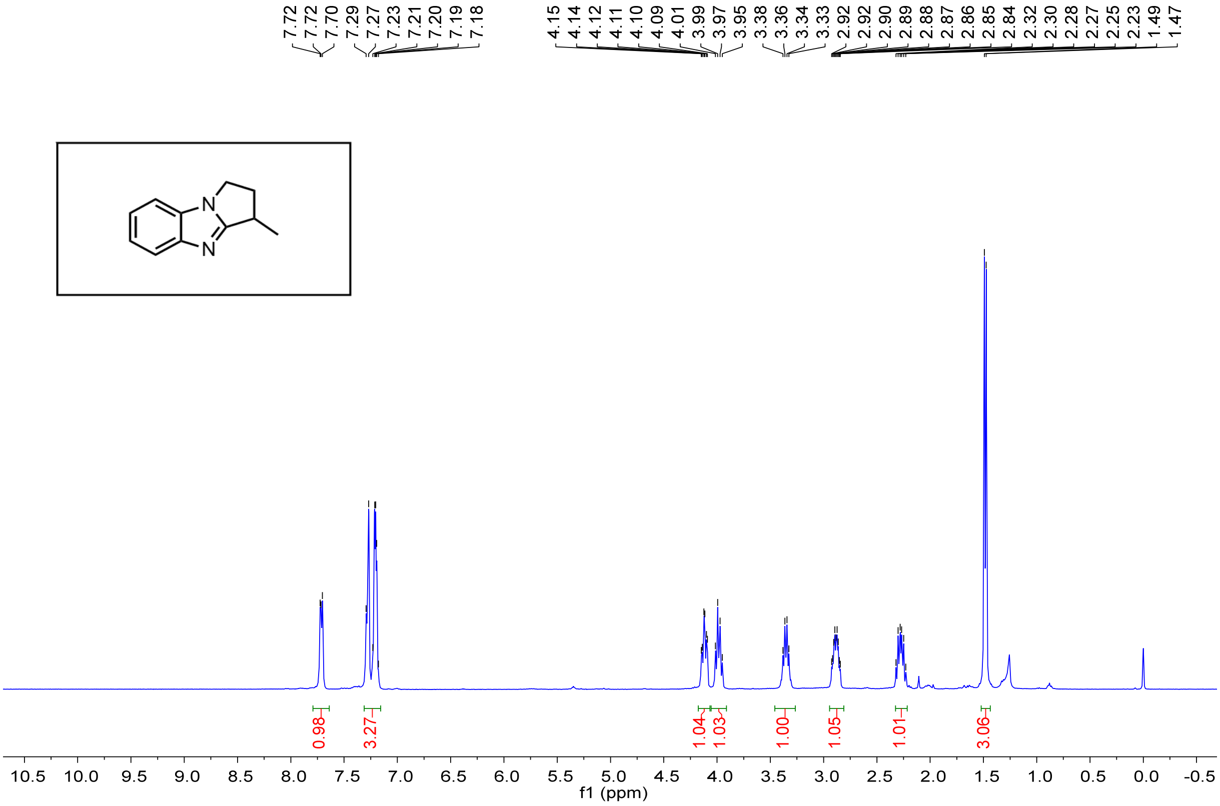

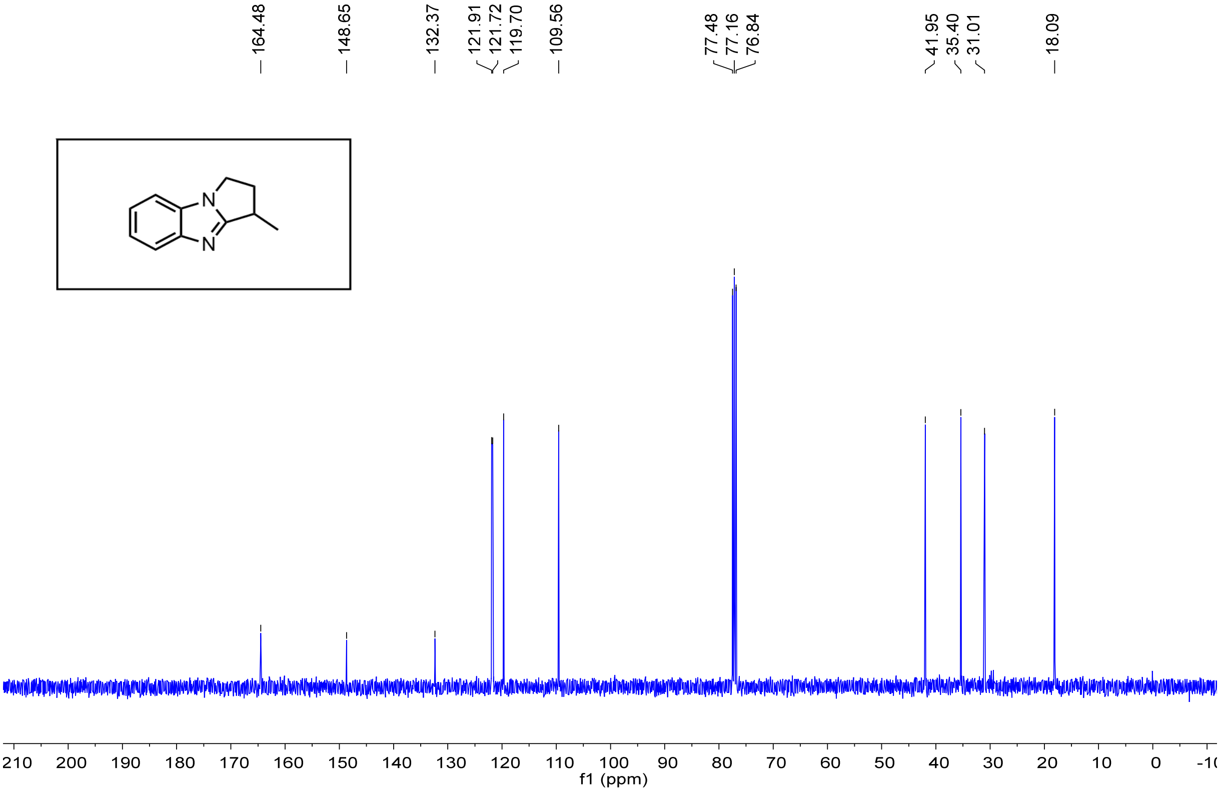

^{1}H (**2a**) and ^{13}C (**2a**) NMR spectra

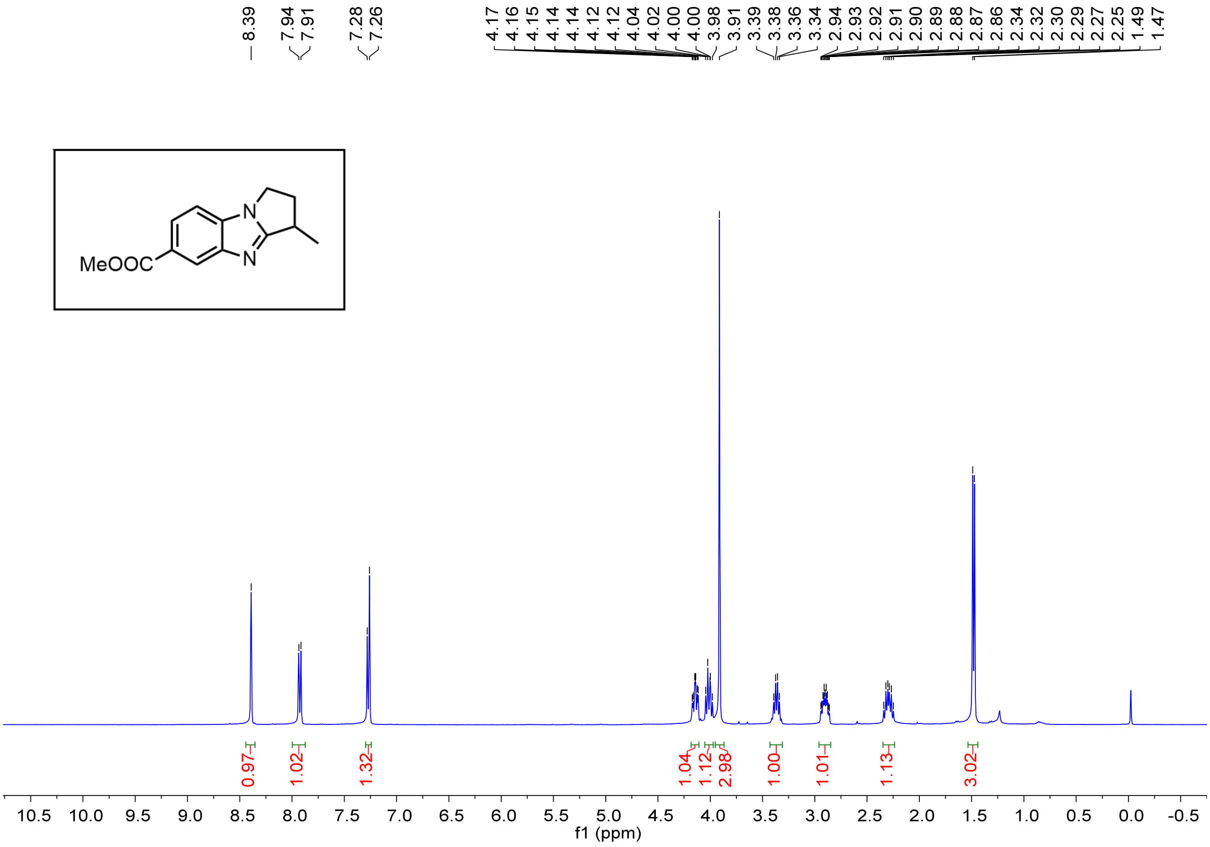

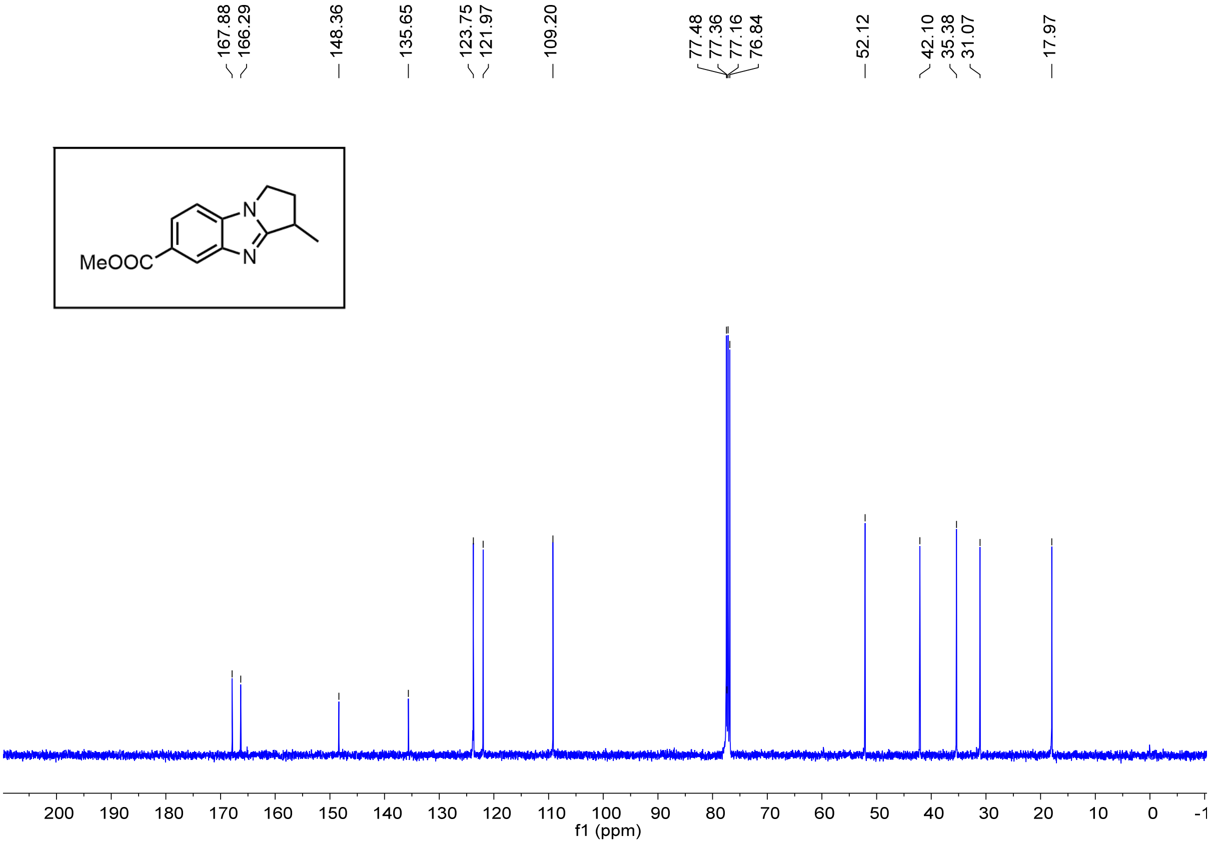

^{1}H (**2b**) and ^{13}C (**2b**) NMR spectra

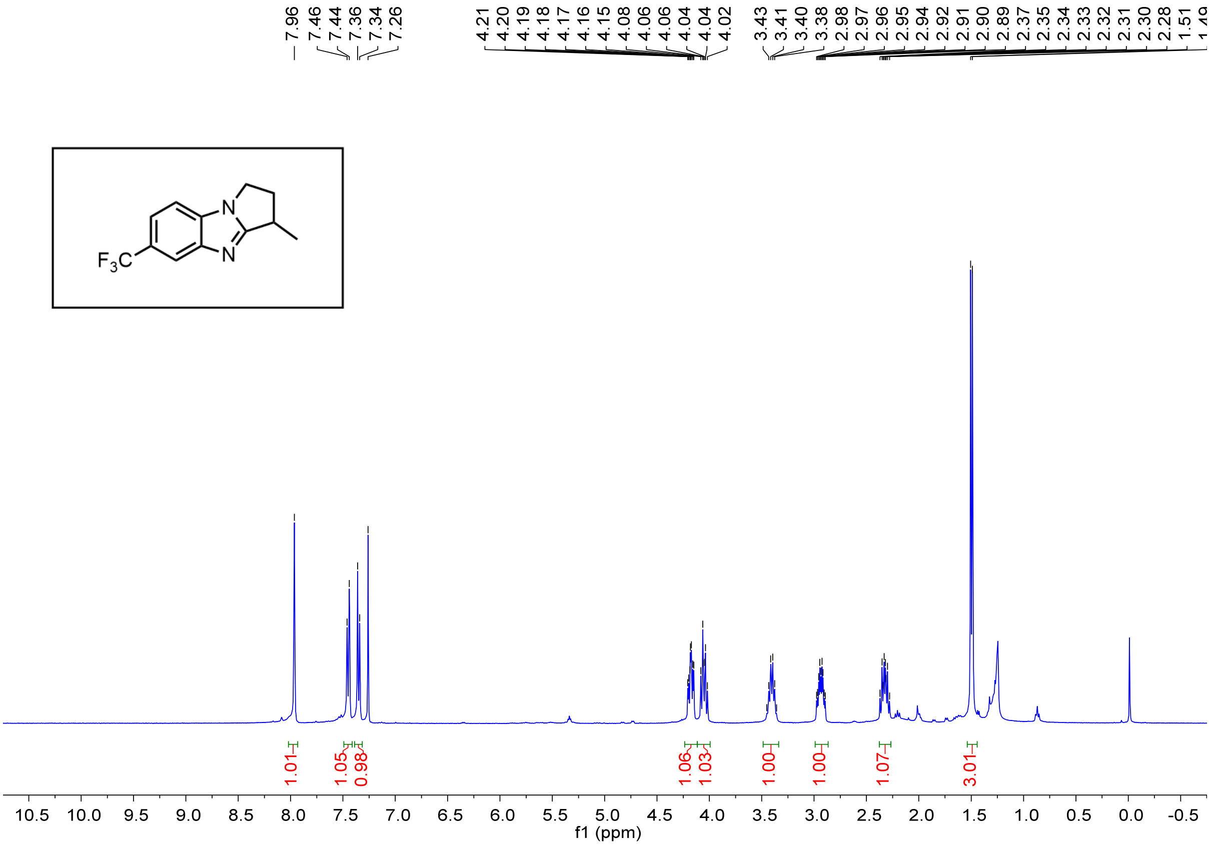

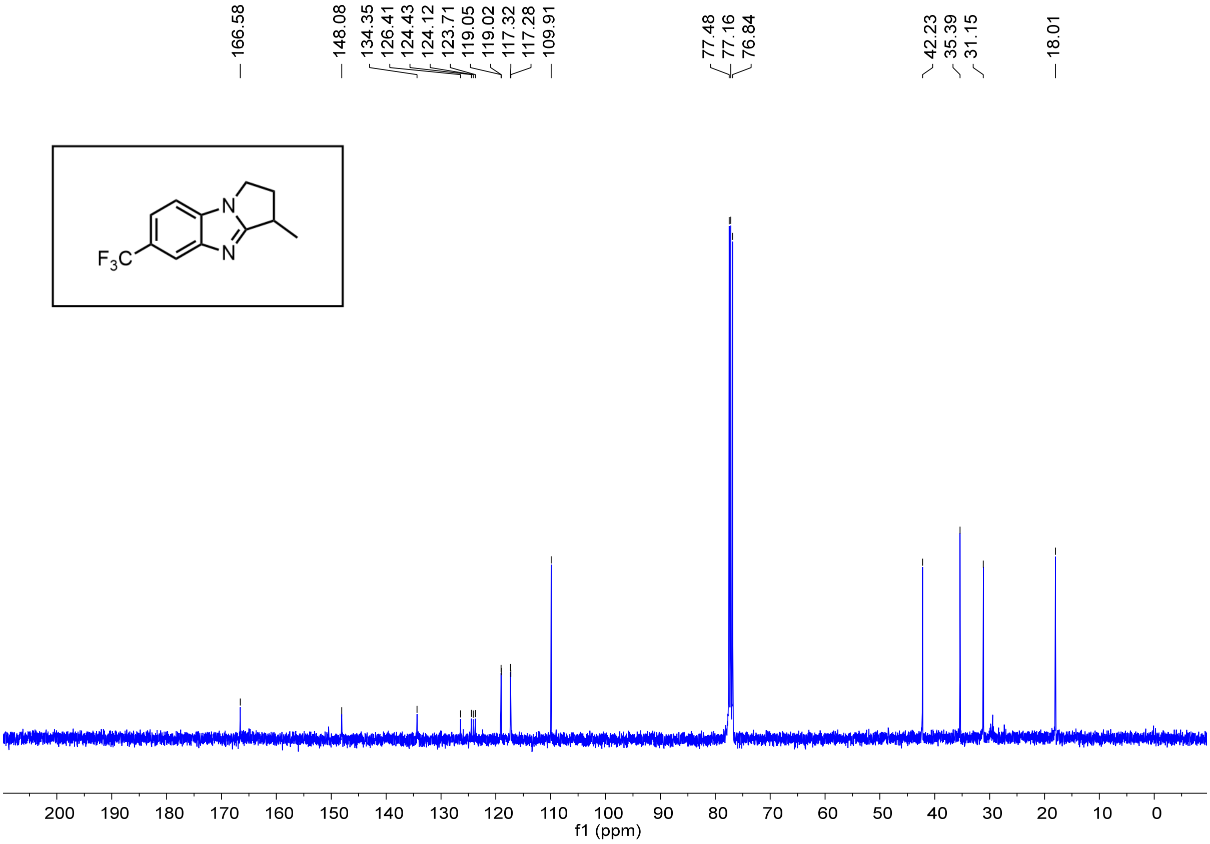

^{1}H (**2c**) and ^{13}C (**2c**) NMR spectra

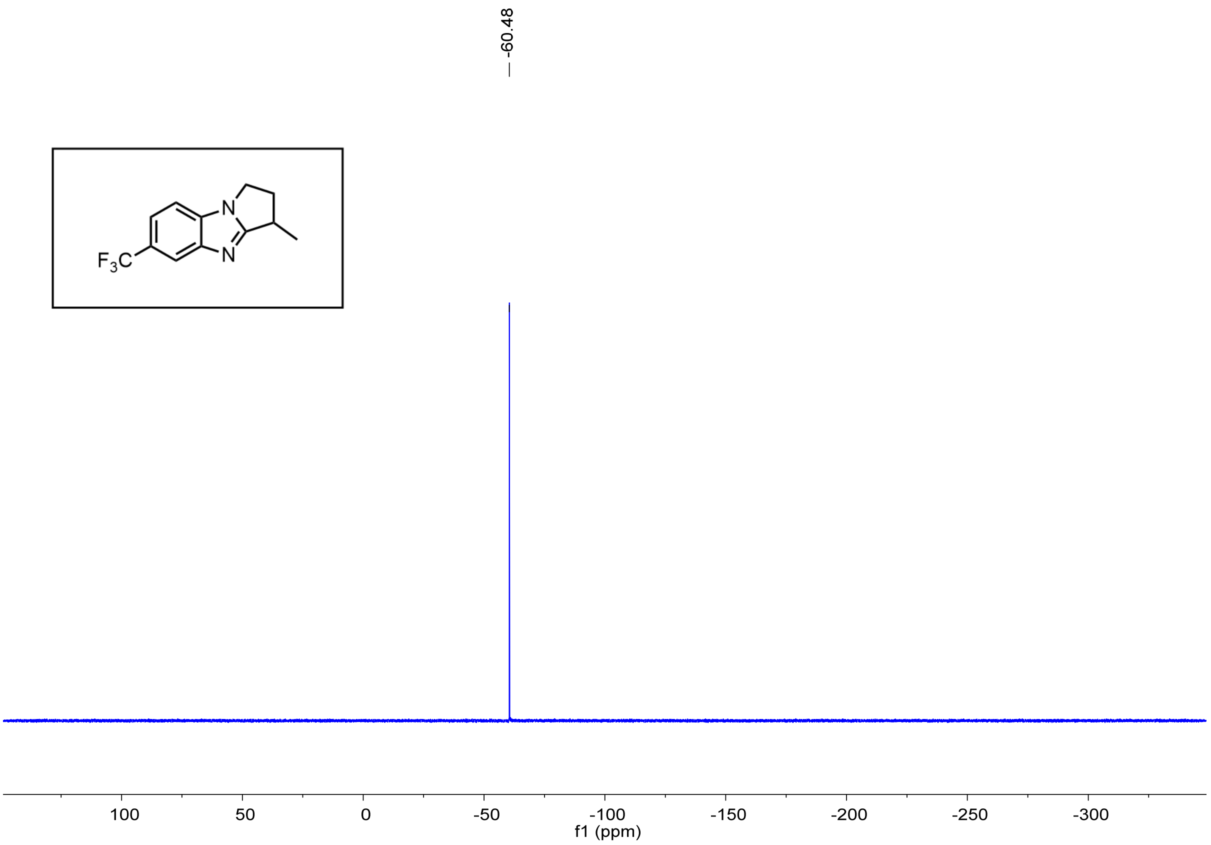

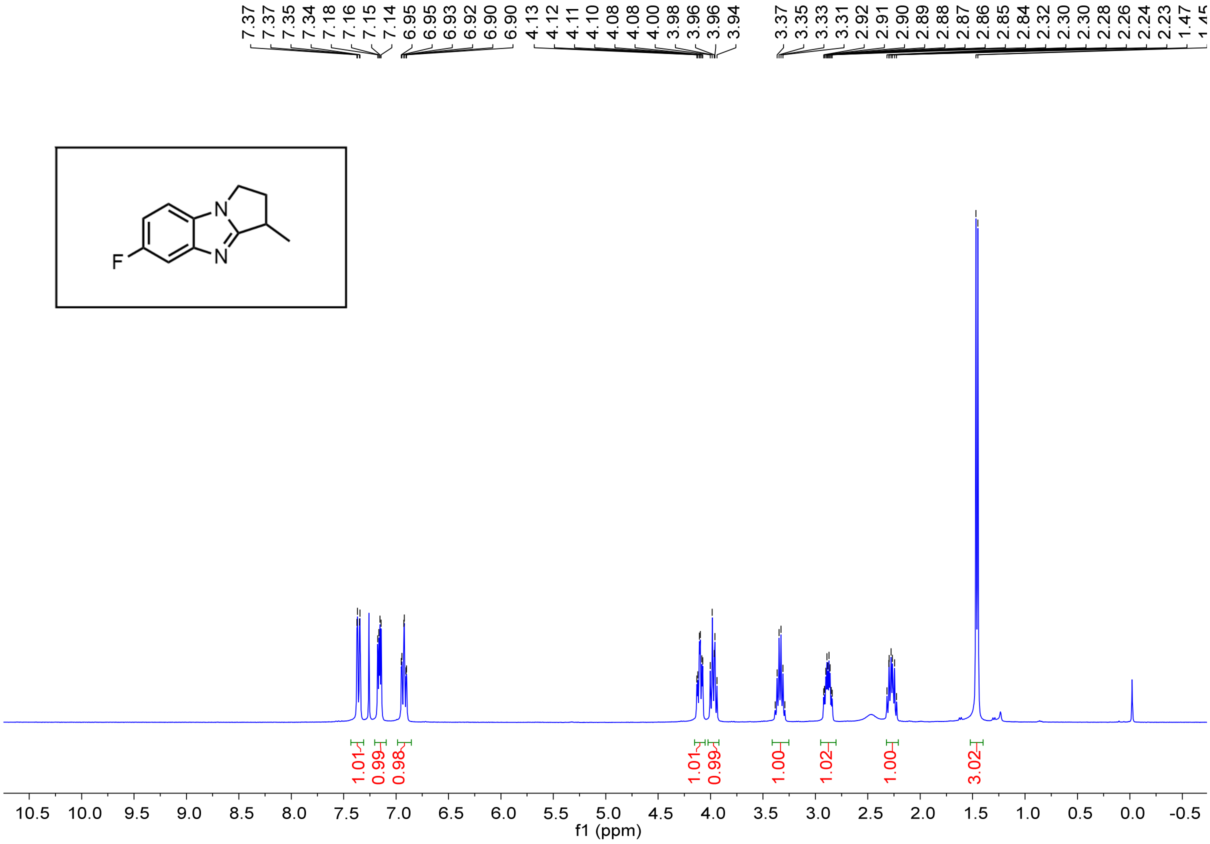

^{19}F (**2c**) and ^{1}H (**2d**) NMR spectra

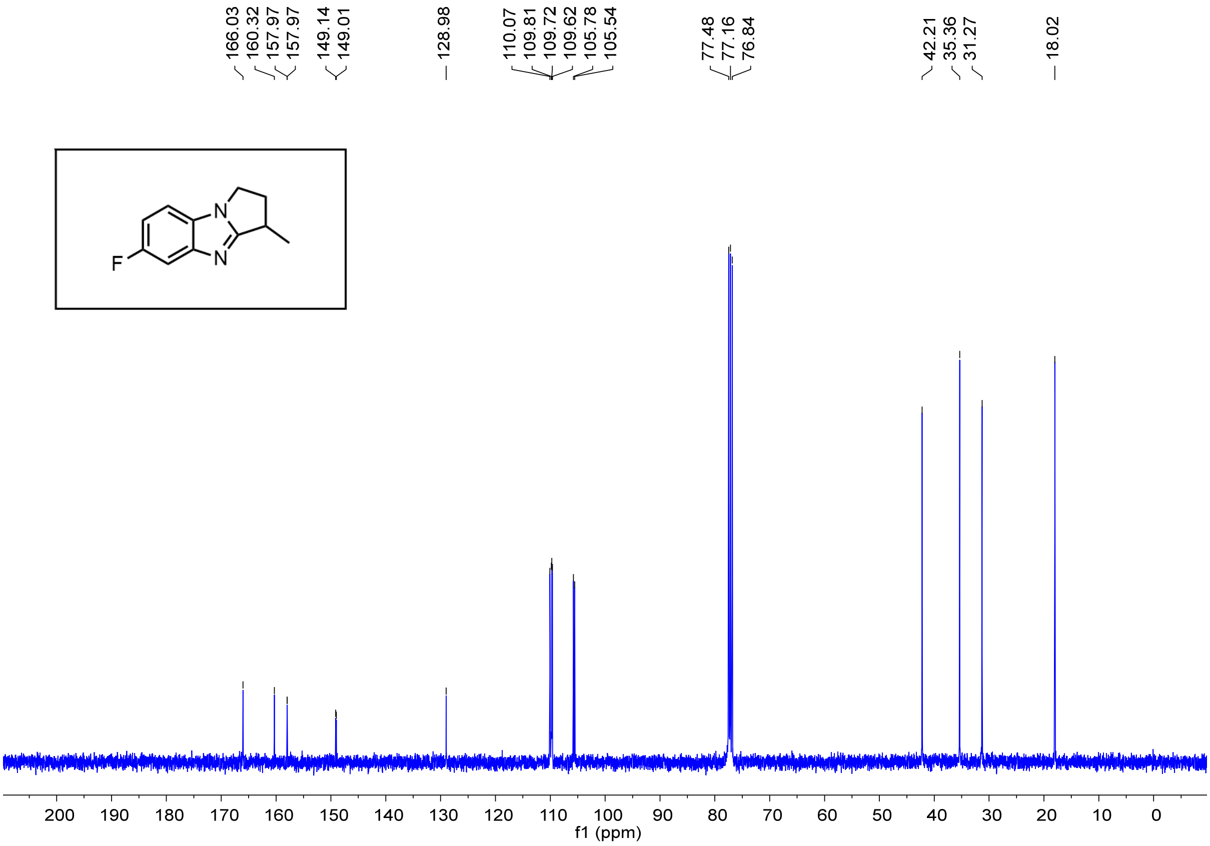

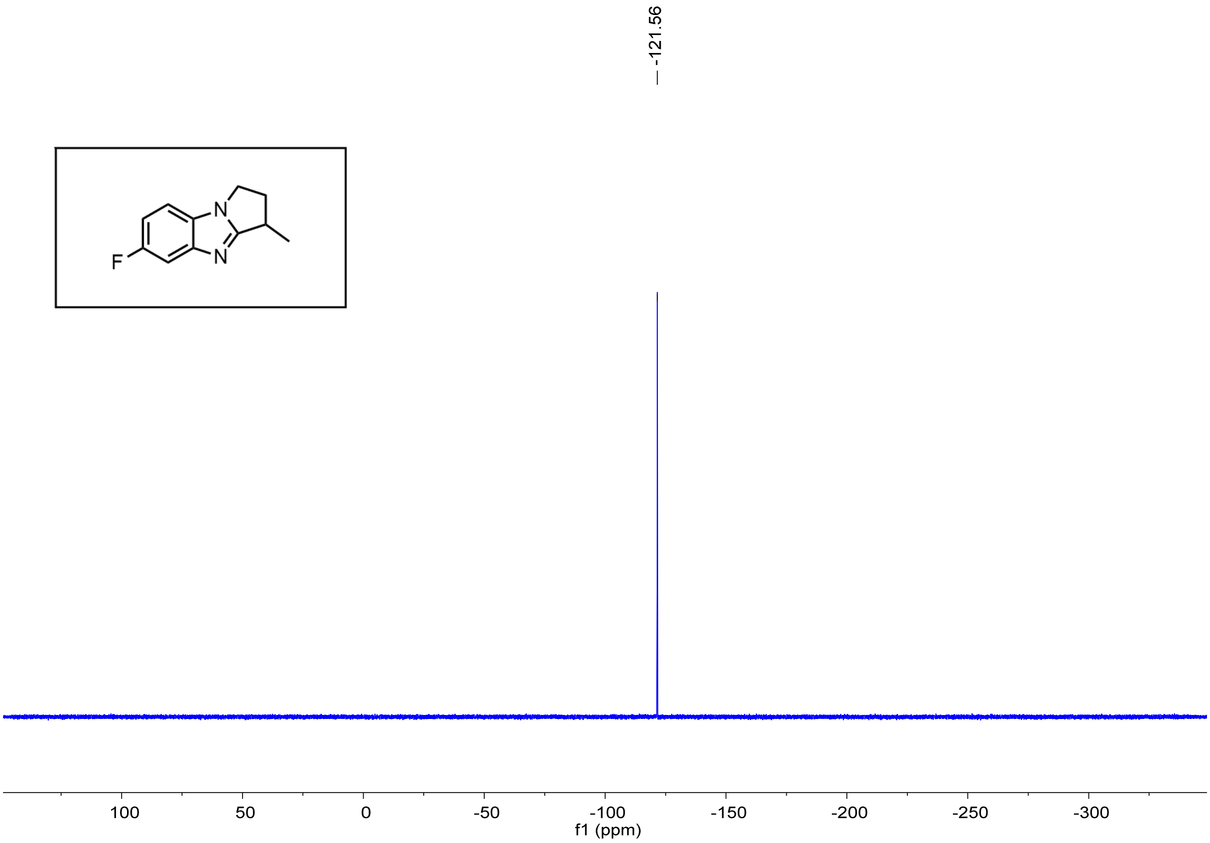

^{13}C (**2d**) and ^{19}F (**2d**) NMR spectra

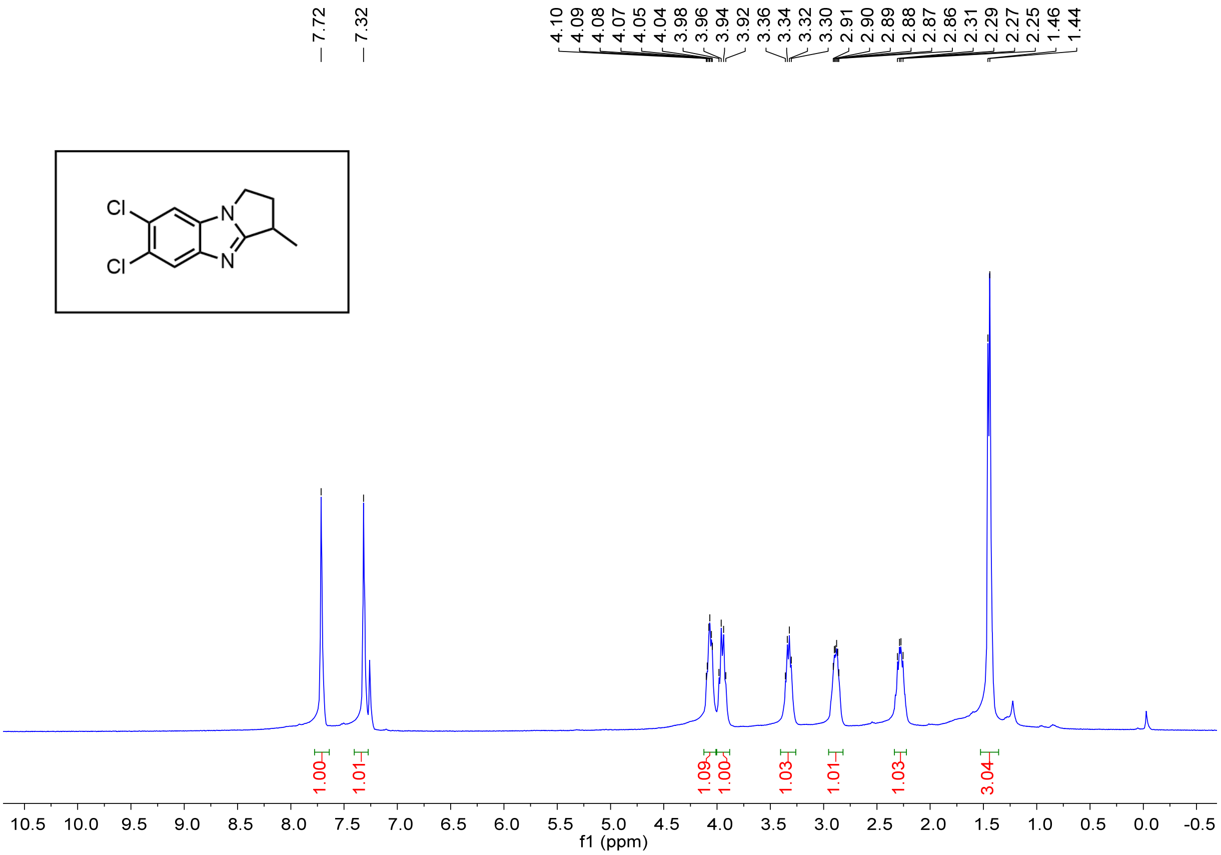

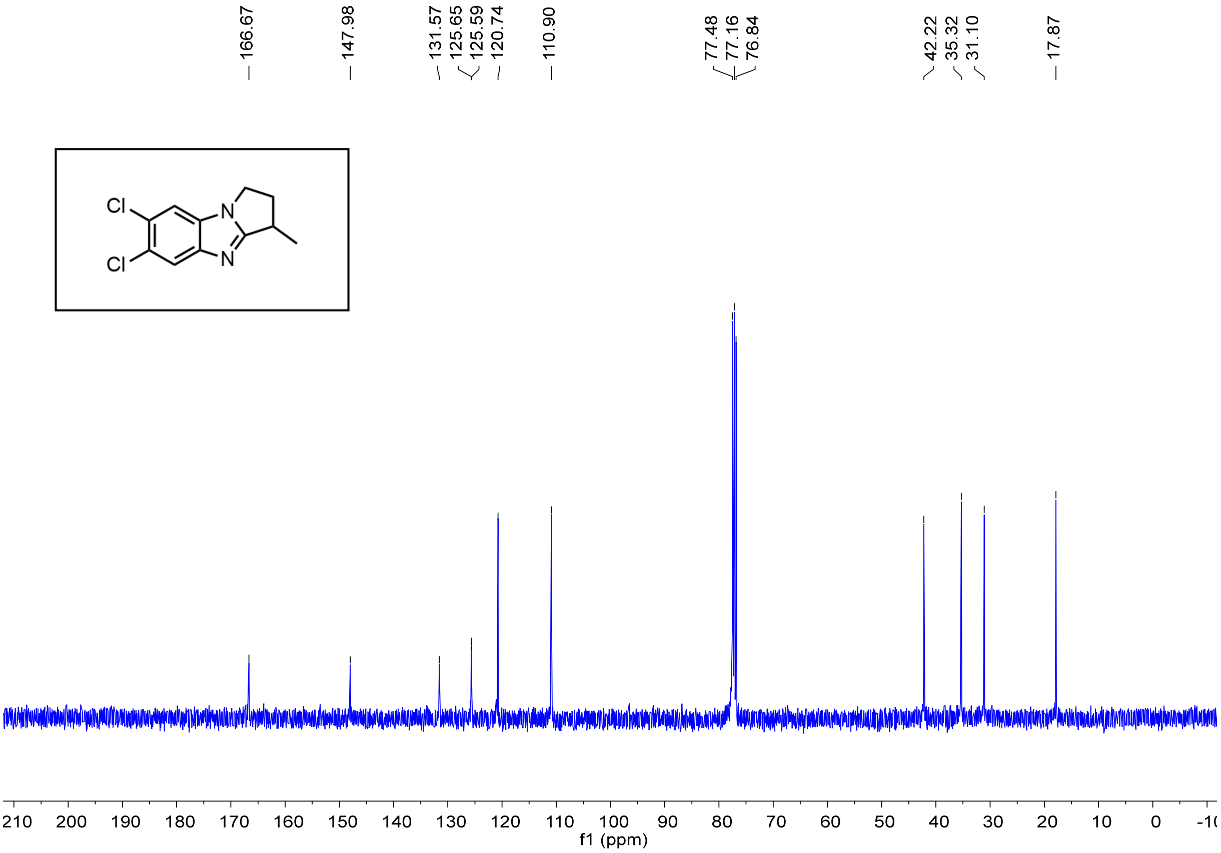

^{1}H (**2e**) and ^{13}C (**2e**) NMR spectra

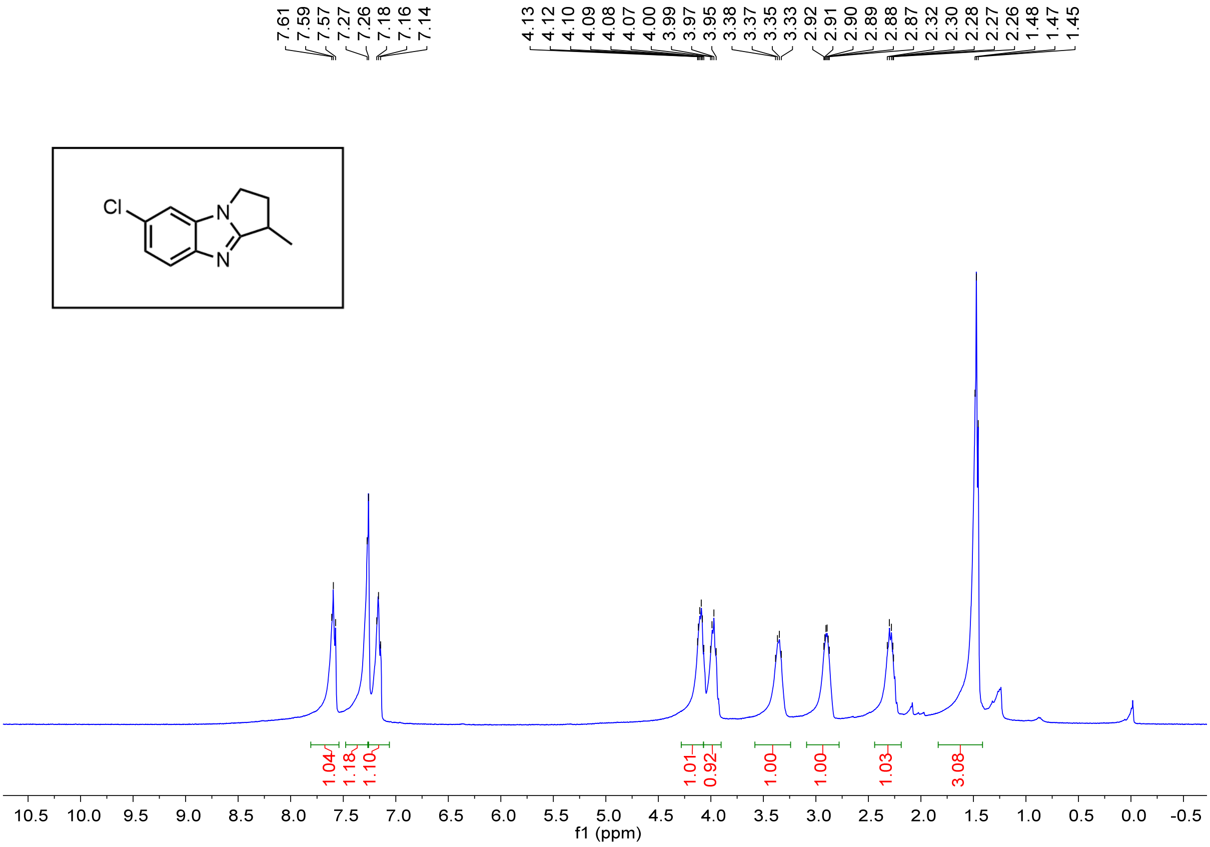

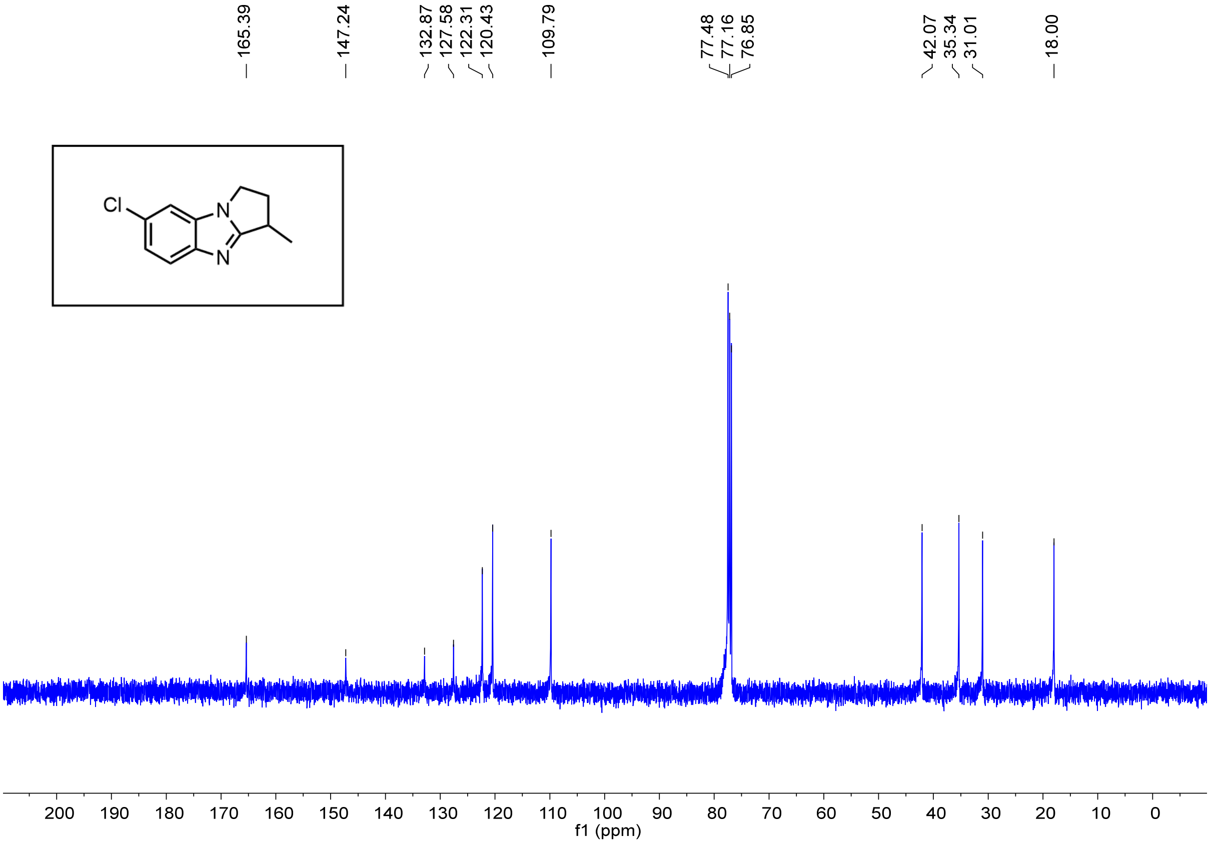

^{1}H (**2f**) and ^{13}C (**2f**) NMR spectra

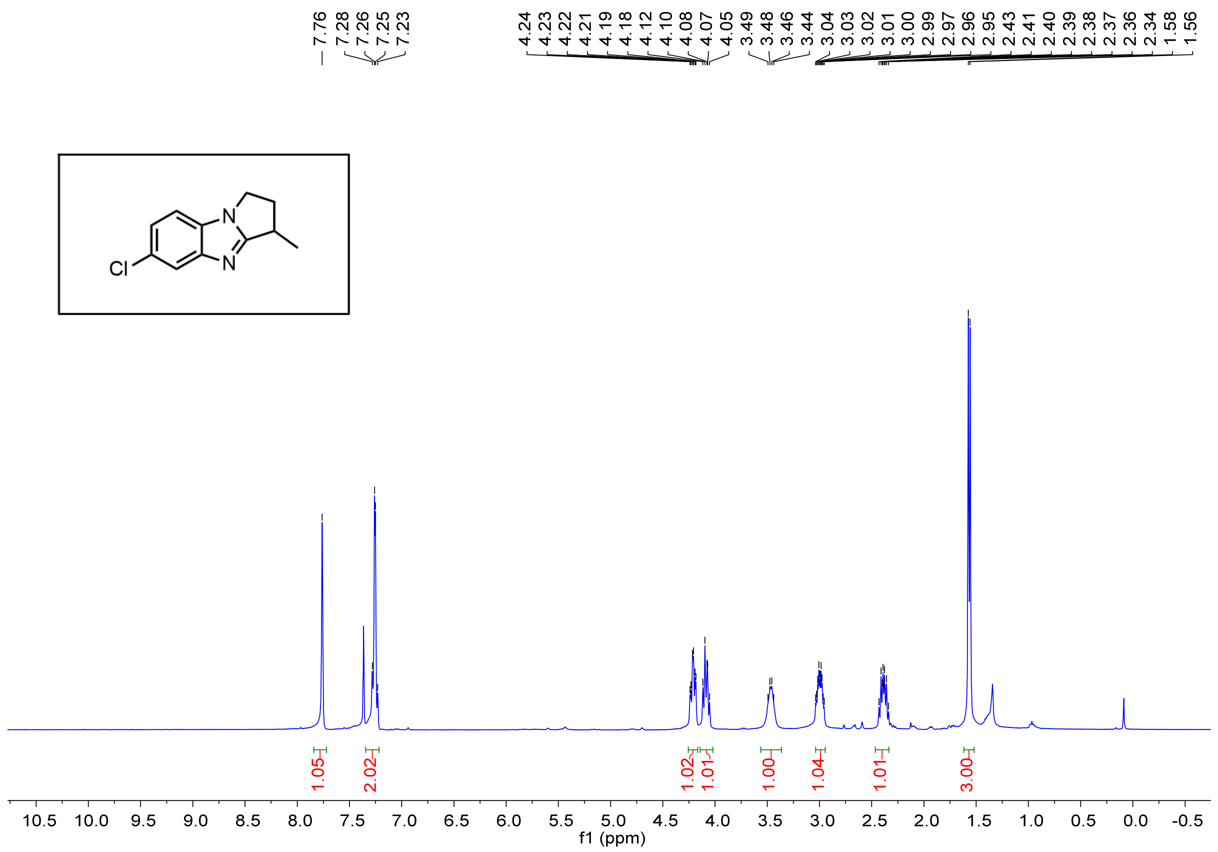

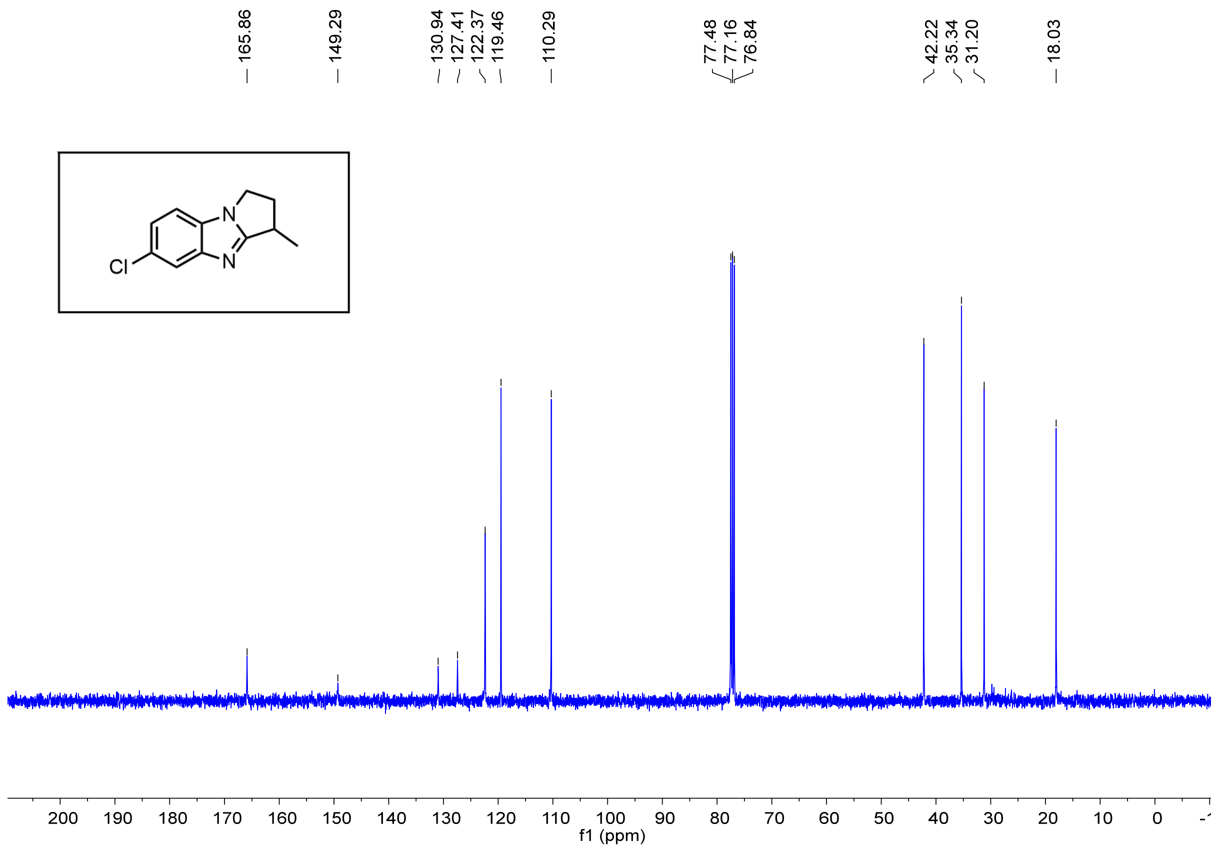

^{1}H (**2g**) and ^{13}C (**2g**) NMR spectra

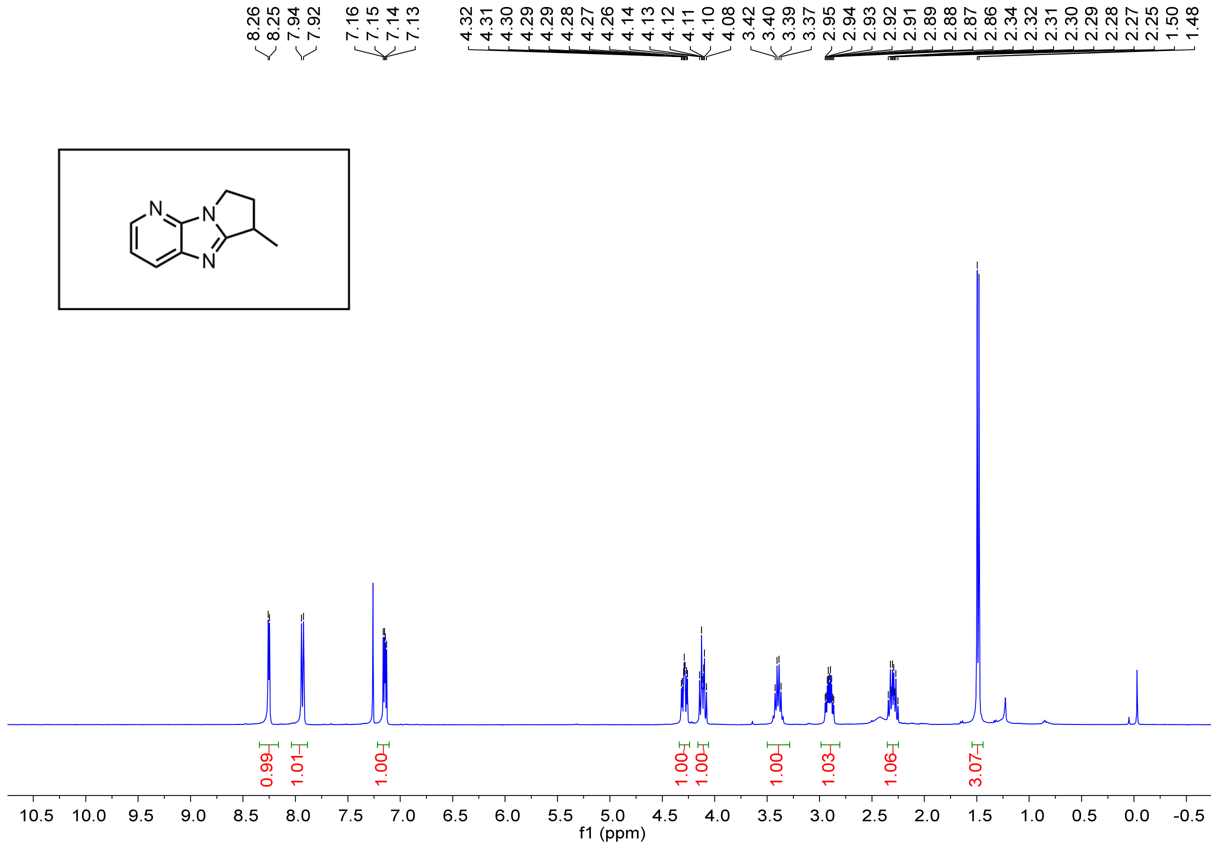

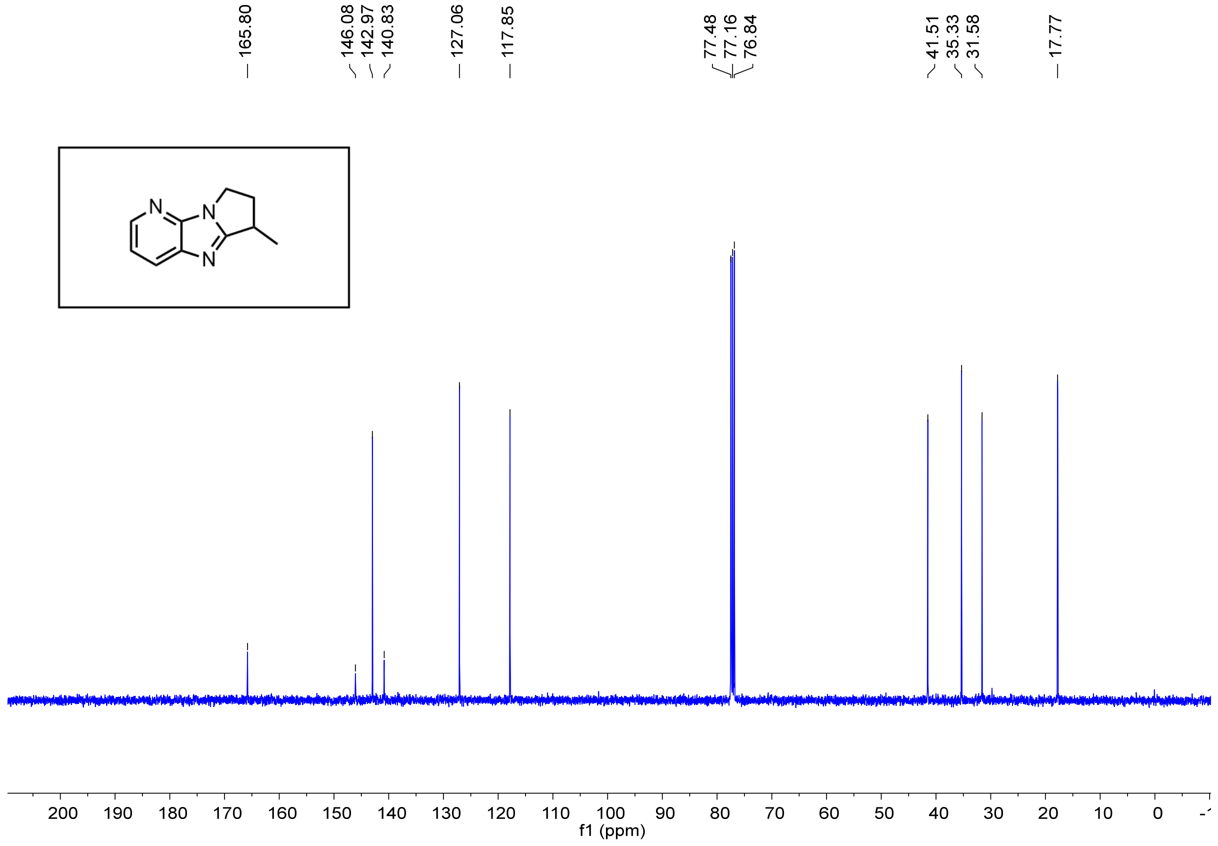

^{1}H (**2h**) and ^{13}C (**2h**) NMR spectra

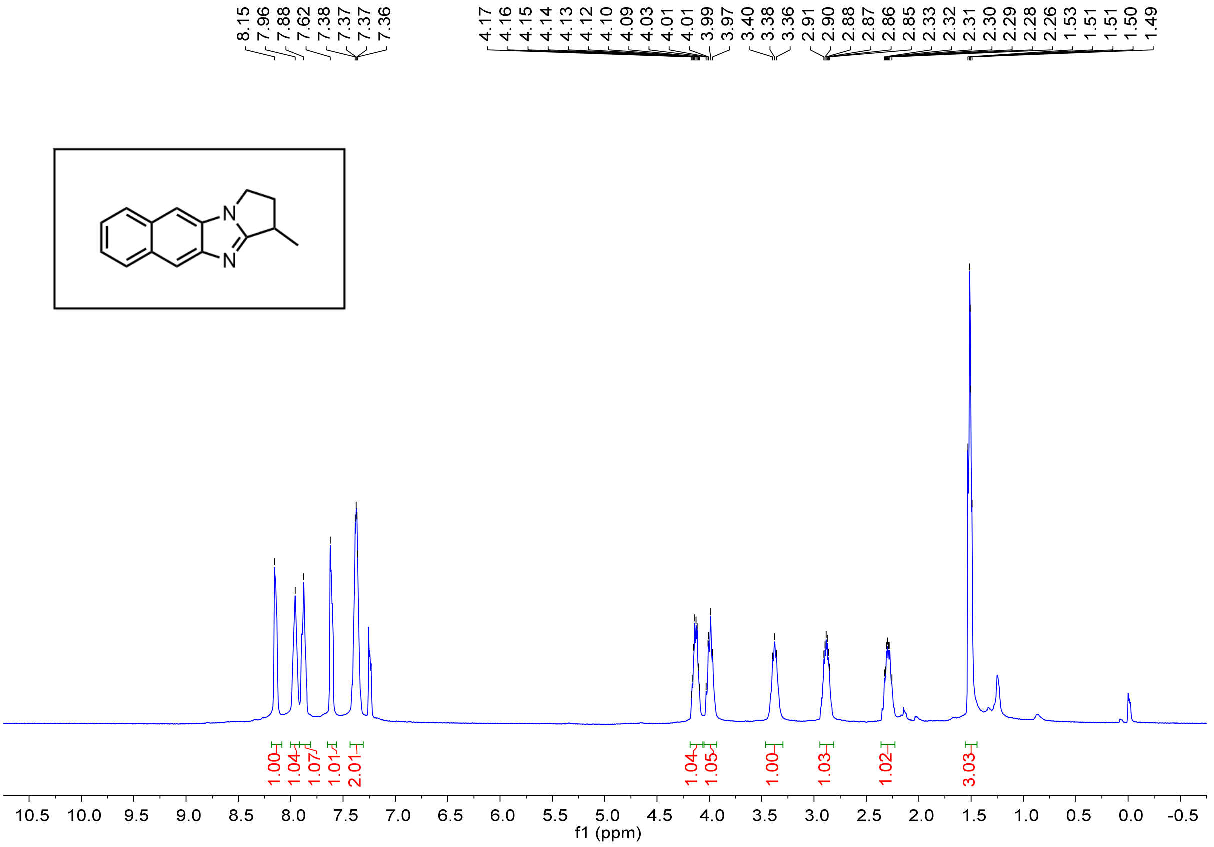

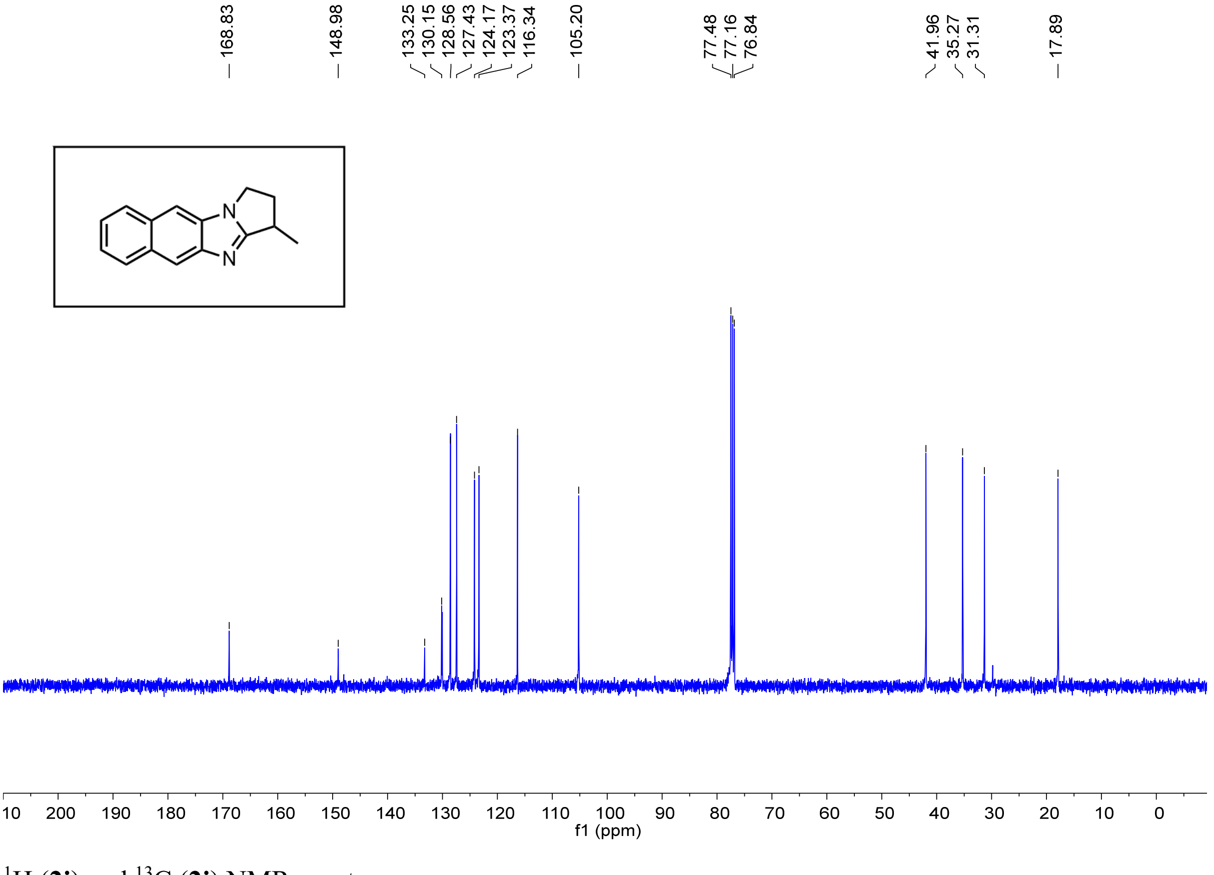

^{1}H (**2i**) and ^{13}C (**2i**) NMR spectra

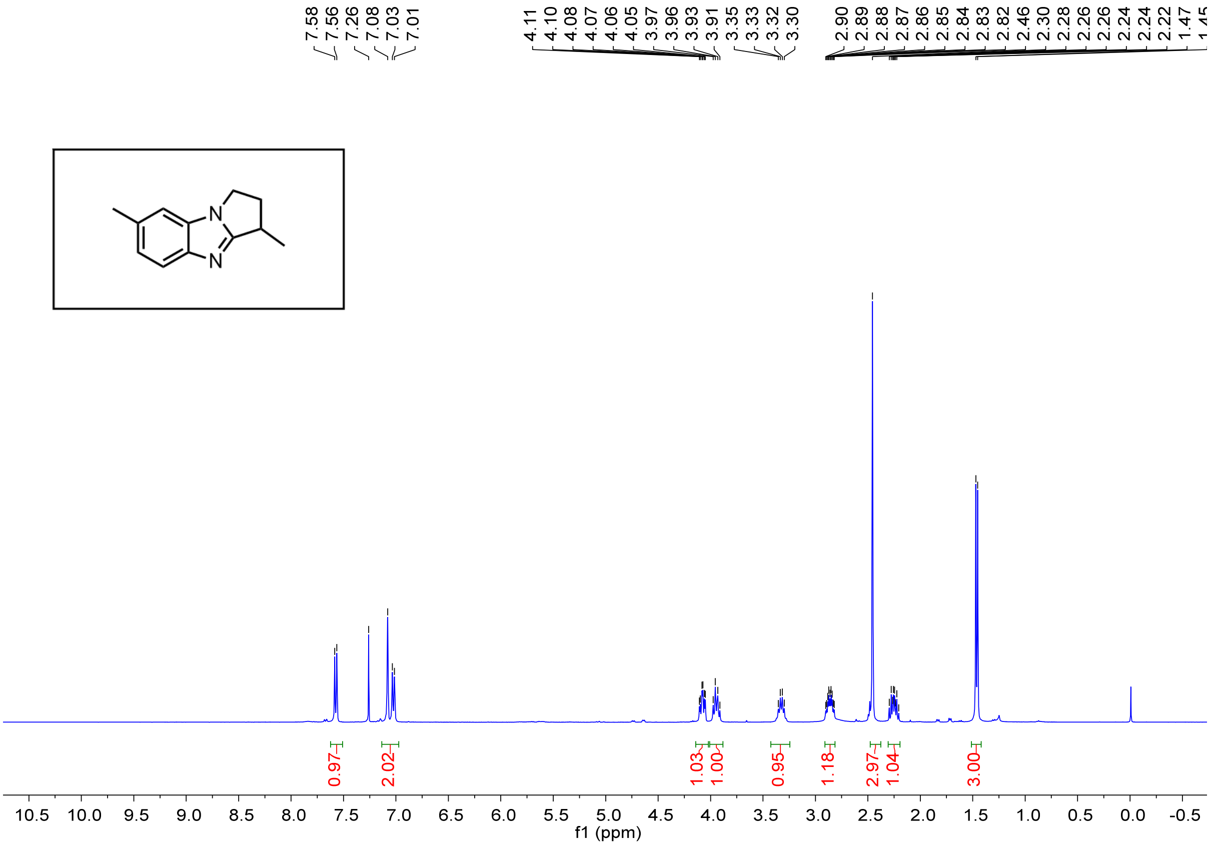

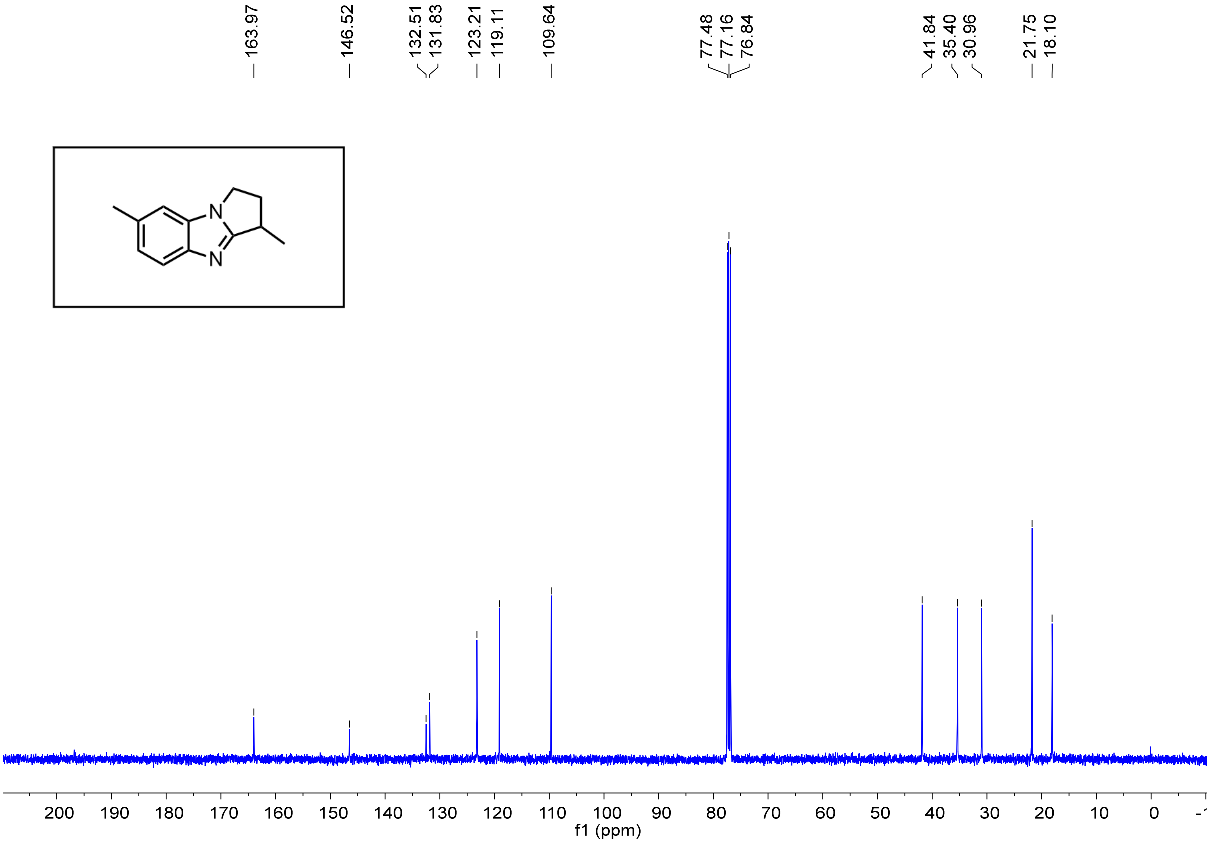

^{1}H (**2j**) and ^{13}C (**2j**) NMR spectra

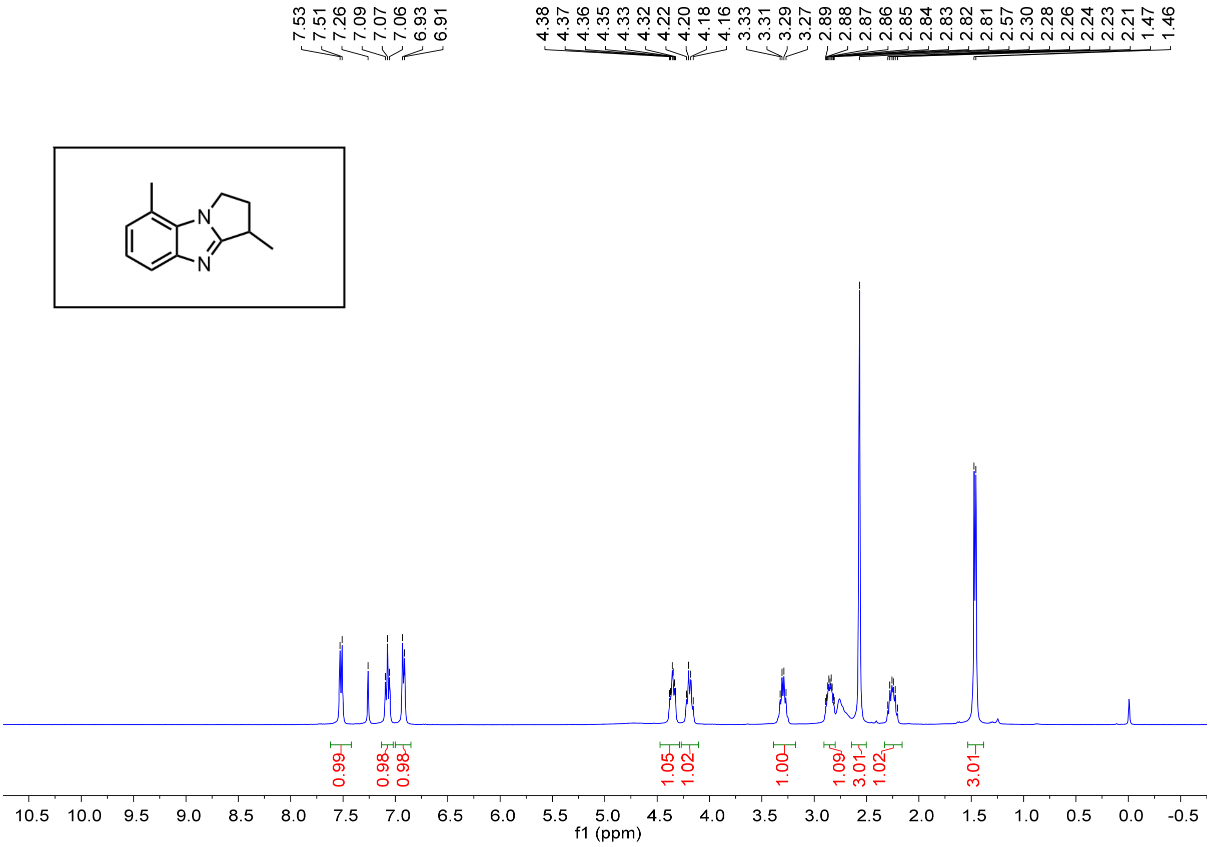

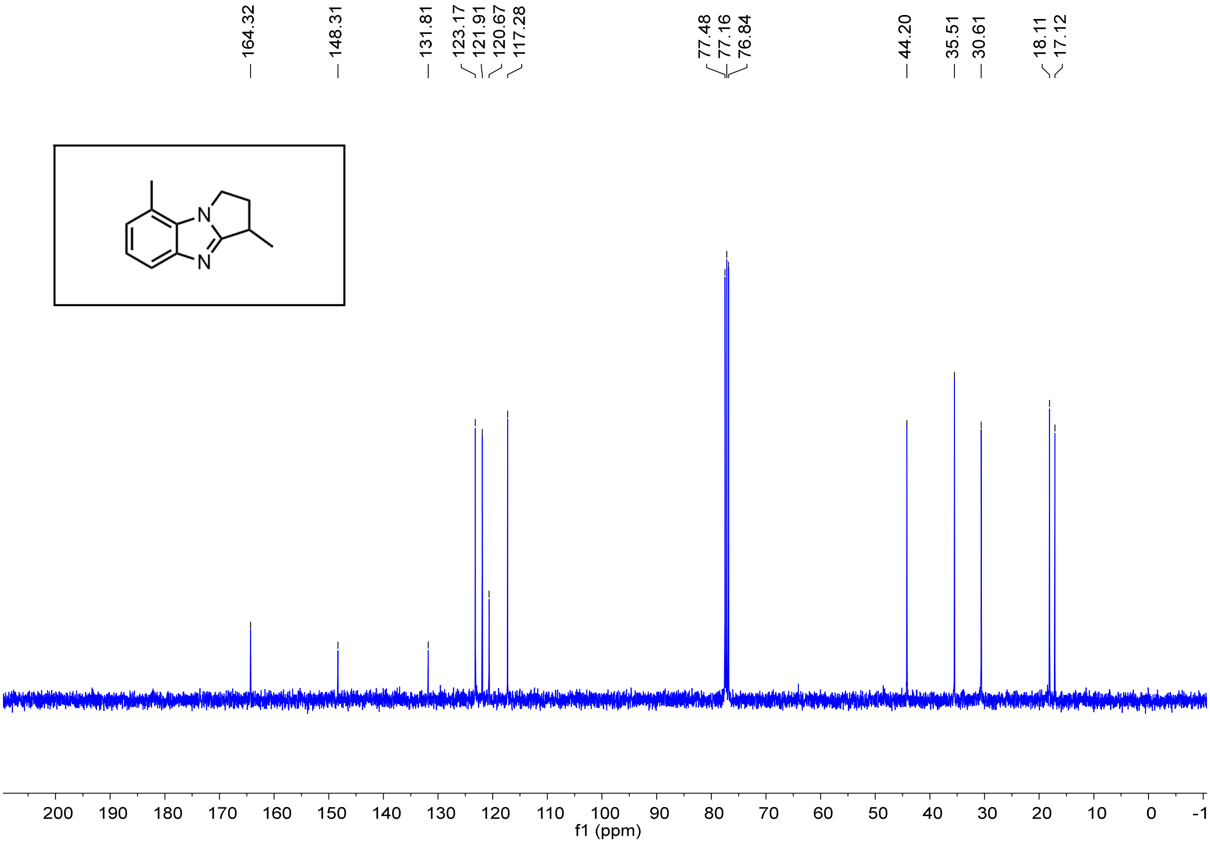

^{1}H (**2k**) and ^{13}C (**2k**) NMR spectra

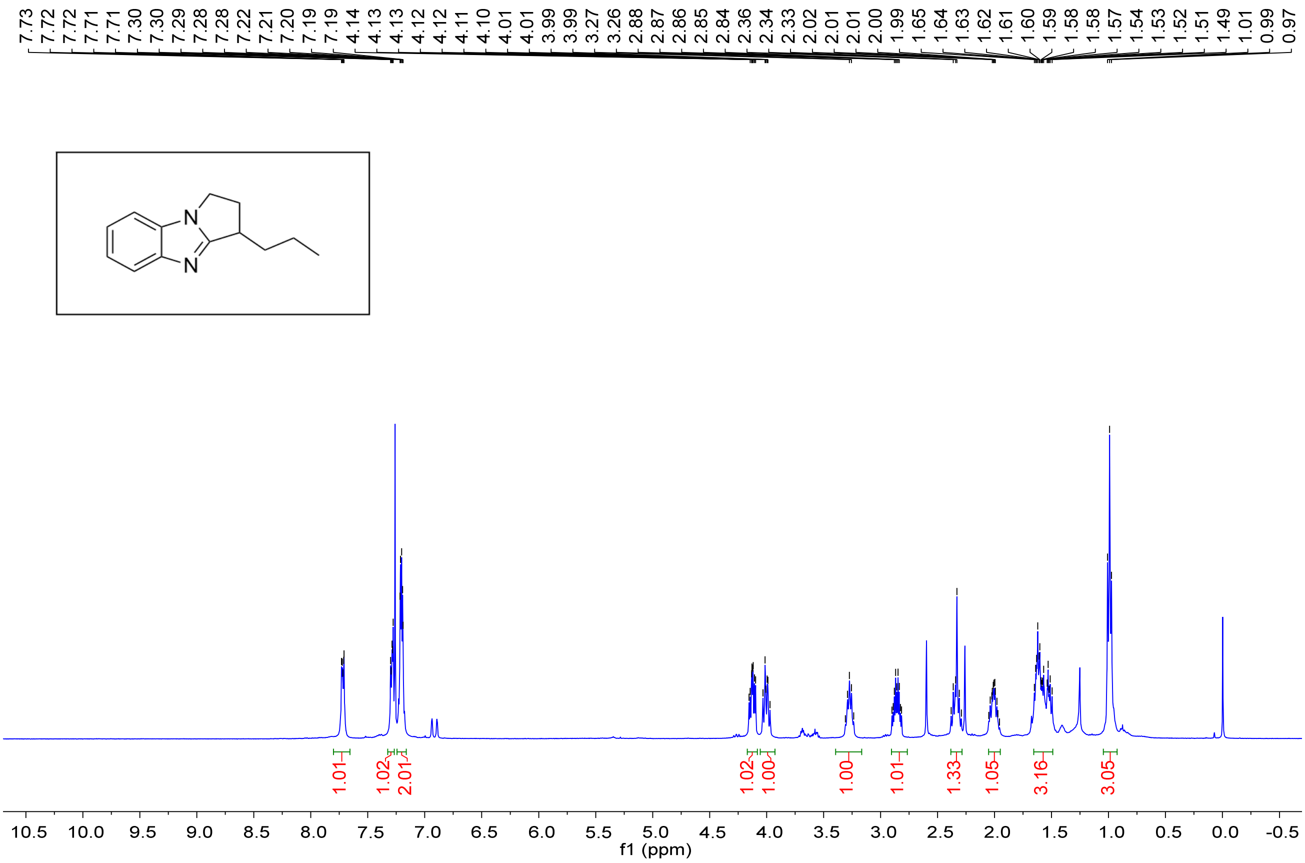

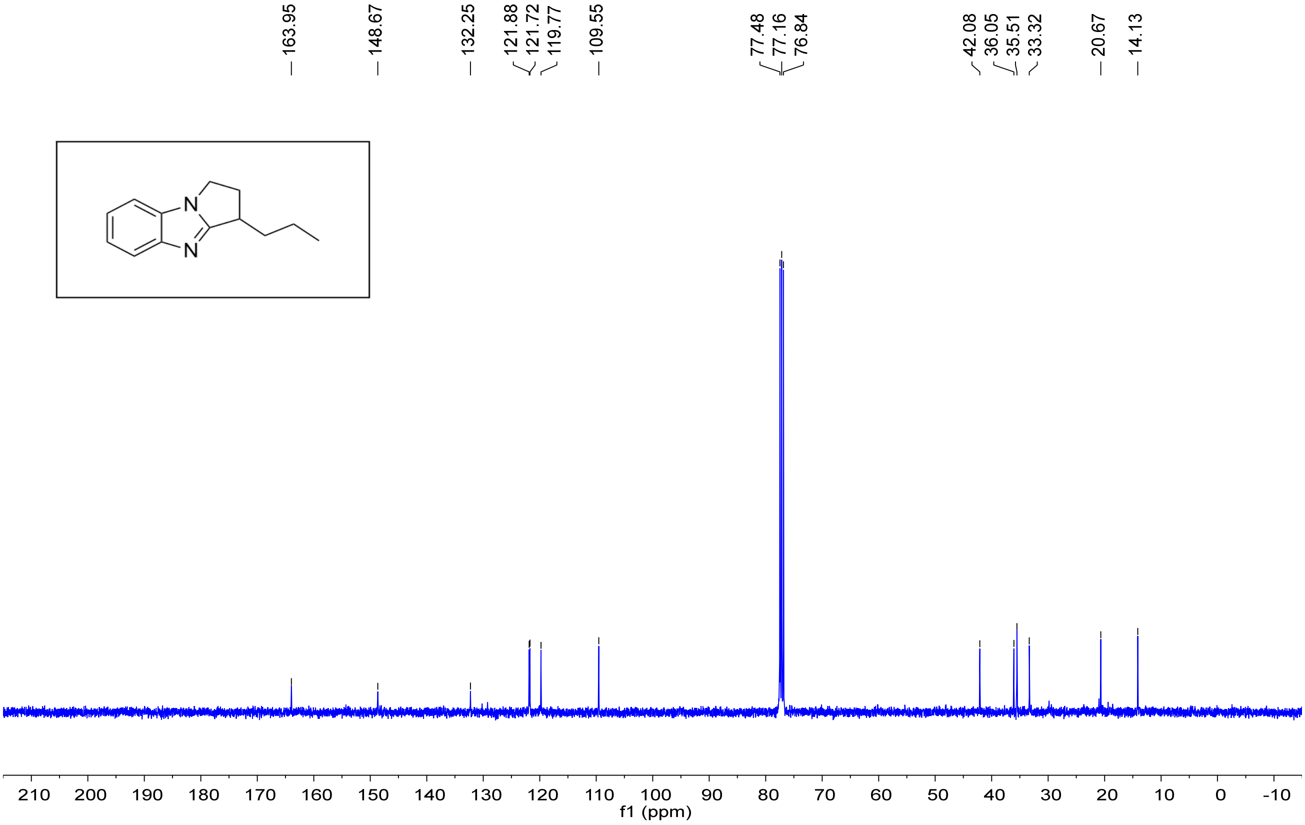

^{1}H (**2l**) and ^{13}C (**2l**) NMR spectra

7. HPLC Charts of Chiral Products

Racemic sample:

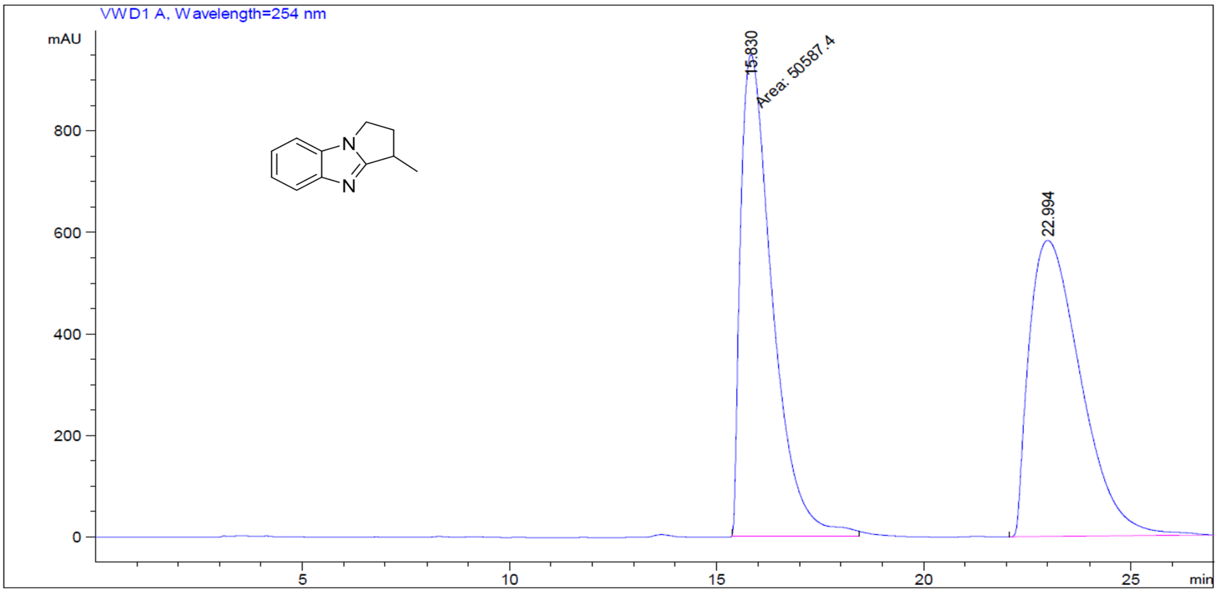

Peak #	RetTime [min]	Type	Width [min]	Area [mAU*s]	Height [mAU]	Area %
1	15.830	MM	0.8888	5.05874e4	948.60620	49.9510
2	22.994	BBA	1.3465	5.06866e4	583.46539	50.0490

Rh catalysis:

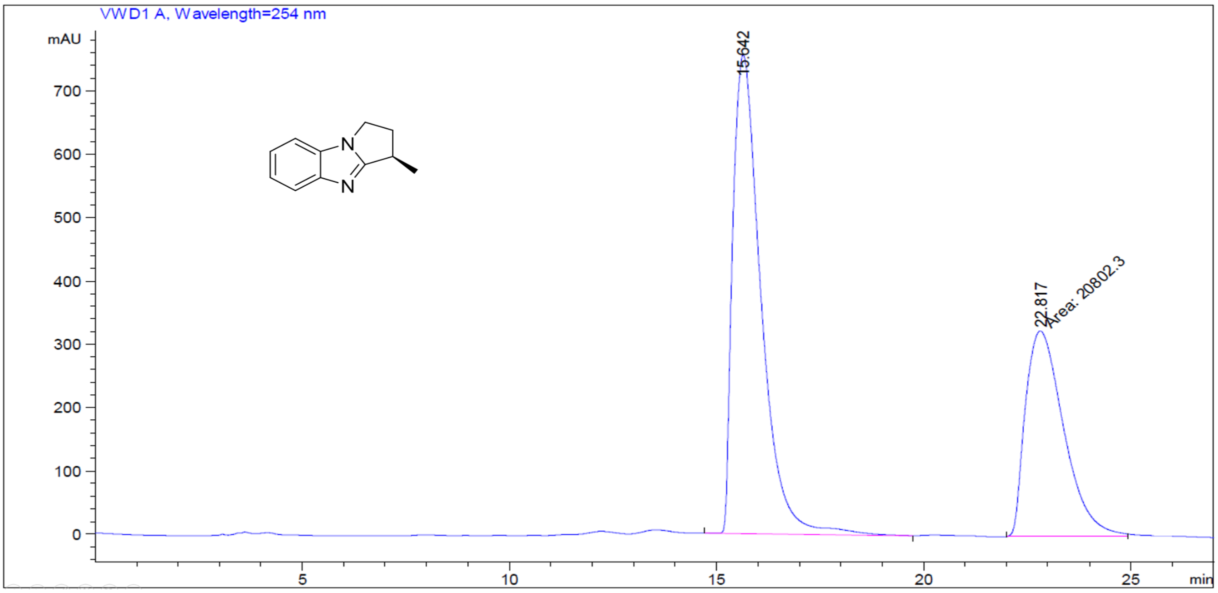

Peak #	RetTime [min]	Type	Width [min]	Area [mAU*s]	Height [mAU]	Area %
1	15.642	BB	0.7403	3.59138e4	755.91711	63.3220
2	22.817	MM	1.0706	2.08023e4	323.83817	36.6780

Racemic sample:

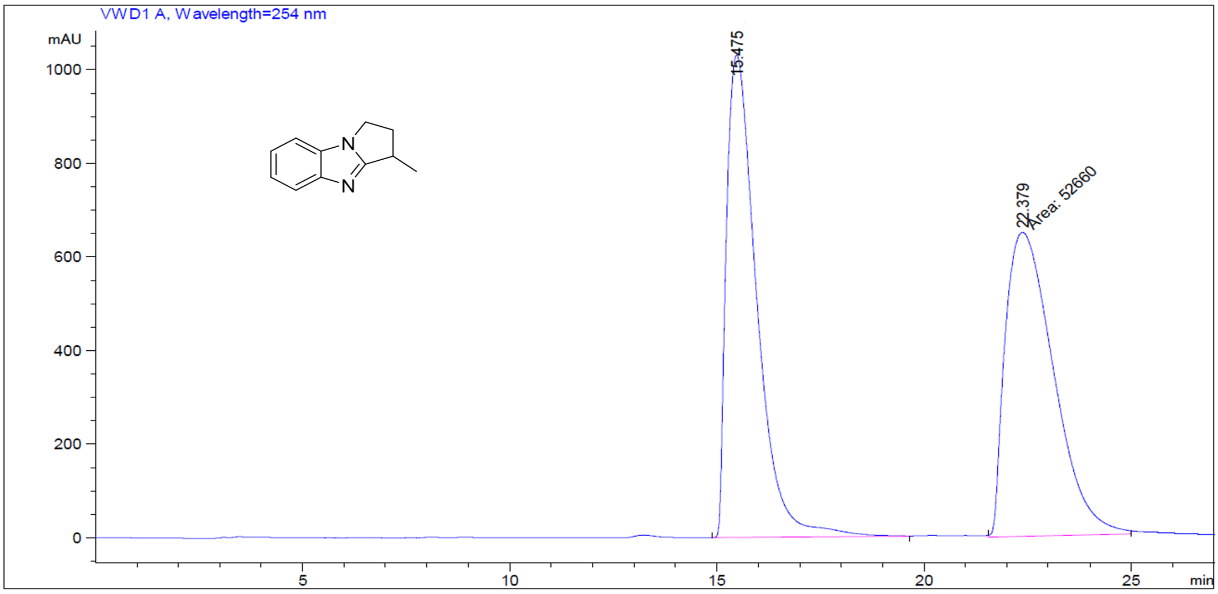

Peak #	RetTime [min]	Type	Width [min]	Area [mAU*s]	Height [mAU]	Area %
1	15.475	BB	0.7762	5.23238e4	1030.82886	49.8399
2	22.379	MM	1.3508	5.26600e4	649.74933	50.1601

Co catalysis:

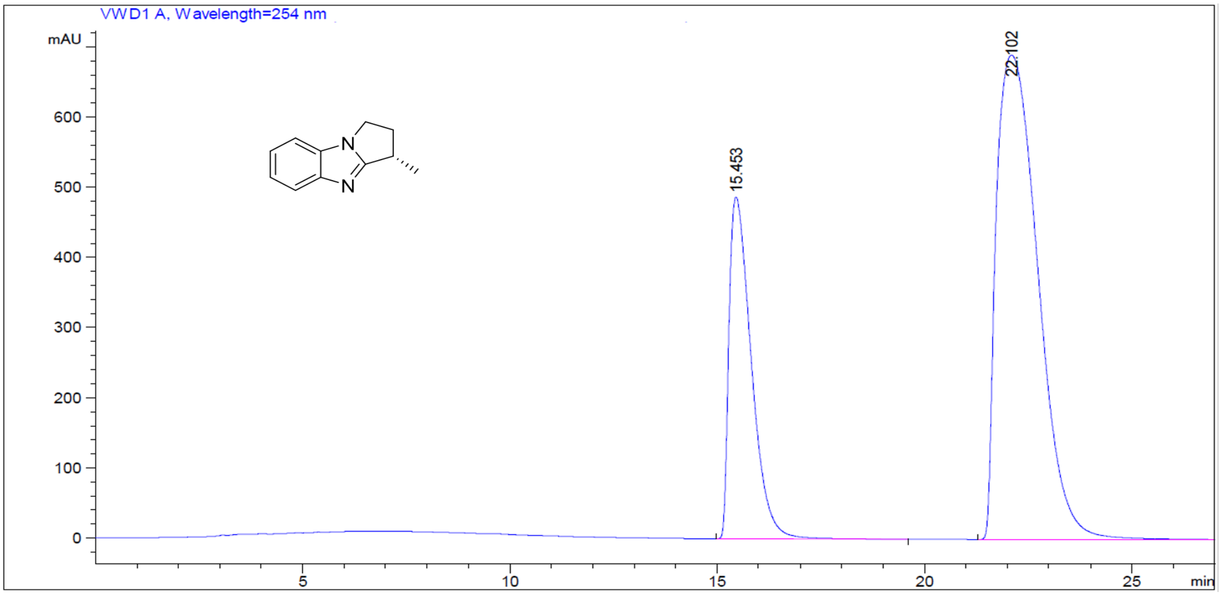

Peak #	RetTime [min]	Type	Width [min]	Area [mAU*s]	Height [mAU]	Area %
1	15.453	BB	0.5773	1.81865e4	487.40973	27.4929
2	22.102	BBA	1.1384	4.79635e4	690.51404	72.5071
